# Acylphosphine Route to Colloidal InP Quantum Dots

**DOI:** 10.1021/jacs.5c01305

**Published:** 2025-03-24

**Authors:** Andriy Stelmakh, Georgios Marnieros, Erik Schrader, Georgian Nedelcu, Oleh Hordiichuk, Eduard Rusanov, Ihor Cherniukh, Daniel Zindel, Hansjörg Grützmacher, Maksym V. Kovalenko

**Affiliations:** † Laboratory of Inorganic Chemistry, Department of Chemistry and Applied Biosciences, ETH Zürich, Vladimir Prelog Weg 1, Zürich CH-8093, Switzerland; ‡ Laboratory for Thin Films and Photovoltaics, Empa − Swiss Federal Laboratories for Materials Science and Technology, Überlandstrasse 129, Dübendorf CH-8600, Switzerland; § Laboratory of Physical Chemistry, Department of Chemistry and Applied Biosciences, ETH Zürich, Vladimir Prelog Weg 2, Zürich CH-8093, Switzerland; # SKKU Institute of Energy Science and Technology (SIEST), Sungkyunkwan University (SKKU), 2066, Seobu-ro, Jangan-gu, Suwon, Gyeonggi-do 16419, Republic of Korea

## Abstract

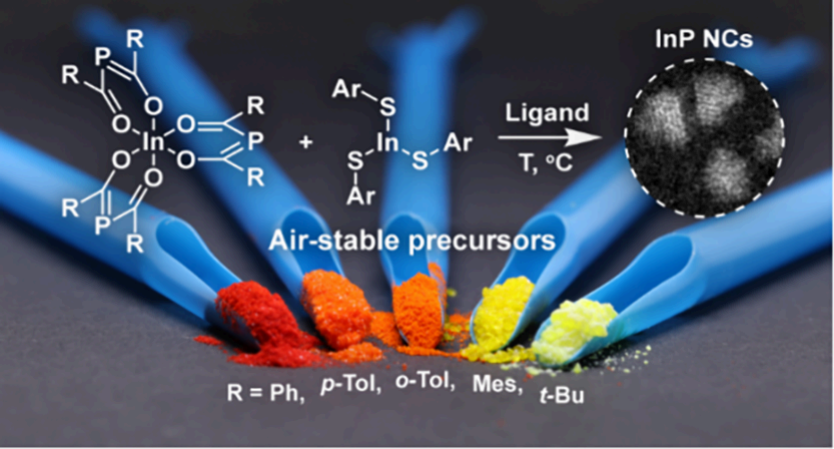

InP-based quantum dots (QDs) represent the major commercial success of colloidal semiconductor nanocrystals (NCs). A combination of the robust, mostly covalent, structure and nontoxic nature of the constituent elements makes them a QD material of choice for display and LED technologies. Despite successful commercial realization, InP NCs still lack synthetic versatility and robustness, seen, for instance, as a continued quest to substitute a commonly used pyrophoric and expensive tris(trimethylsilyl)phosphine precursor. Herein, we propose solid-state, nonpyrophoric, and synthetically readily accessible acylphosphines as convenient phosphorus precursors for the synthesis of InP NCs. When combined with suitable anionic nucleophiles, such as arylthiolates, both tris(acyl)phosphines and indium complexes of bis(acyl)phosphines act as efficient sources of the P^3–^ anion, as corroborated by NMR spectroscopy and powder X-ray diffraction studies. This type of reactivity is utilized in colloidal synthesis of uniform InP QDs with well-defined excitonic features in their optical absorption spectra, spanning 460–600 nm. The conversion kinetics and therefore the final NC size are controlled by the nature of acyl substituents and by the use of either indium or zinc long-chain carboxylates as ligands. The proposed acylpnictide route is anticipated to foster the development of other metal phosphide and metal arsenide NCs.

## Introduction

With the bulk bandgap of 1.35 eV and the Bohr exciton radius of *ca*. 10 nm, InP is a popular heavy-metal-free quantum dot (QD) material, offering narrowband and efficient emission in the visible and near-infrared spectral regions.^1−3^ Ventures into the colloidal synthesis of InP nanocrystals (NCs) began soon after the conception of the organometallic hot-injection synthesis method by Bawendi et al. in the early 90s (for Cd chalcogenide NCs).^4,5^ Two decades later, after numerous editions of the preparative protocols for InP-based core–shell NCs, these QDs have been deployed commercially as green and red primary emitters in LCD displays.^6,7^ Good compliance of InP NCs with regulations on the use of toxic elements in consumer electronics had been decisive for winning the market share. Whereas the quality of InP-based QDs in terms of photoluminescence quantum yield and linewidth has been nearly perfected for the primary green and red emission colors,^8,9^ the available synthetic protocols are often cumbersome (e.g., multistep or involving precursor injection with a precisely controlled speed) and give rather limited control over larger NC sizes and other NC morphologies (e.g., nanorods, nanoplatelets). For comparison, II–VI and IV–VI colloidal semiconductor NCs can be produced in a variety of shapes and sizes, often with a (nearly) atomic precision.^10,11^ The limited synthetic control for InP NCs is partially caused by the inherent challenges of III–V materials, such as more covalent bonding and higher propensity to oxidation. Another factor is a much narrower choice of suited precursors that limits the experimental parametric space for forming the NCs. Thus far, air-sensitive, pyrophoric and expensive tris(trimethylsilyl)phosphine ((TMS)_3_P) had remained the primary choice for several decades, from the early days (using dehalosilylation reaction)^4^ to presently used protocols (typically, using long-chain indium carboxylates).^9,12−15^ Furthermore, the extremely high reactivity of (TMS)_3_P causes its rapid depletion during the early stages of the reaction and results in a poor control over the reaction kinetics.^16,17^ Several alternatives to (TMS)_3_P were proposed, including more sterically hindered tris(trialkylsilyl)-^18−20^ and tris(triarylsilyl)phosphines,^21^ tris(trialkylgermyl)phosphines ((R_3_Ge)_3_P),^19,22^ white phosphorus (P_4_),^23^ phosphine gas (PH_3_),^24^ phosphorus trichloride (PCl_3_),^25^ sodium phosphaethynolate (NaOCP),^26^ and various aminophosphines.^27−29^ Of these, inexpensive aminophosphines, such as tris(dimethylamino)- ((DMA)_3_P),^27^ tris(diethylamino)- ((DEA)_3_P)^28^ and tris(pyrazolyl)phosphines ((Pyr)_3_P),^29^ had proven to be the strongest contenders of (TMS)_3_P.^30−32^ The synthesis with aminophoshines is typically conducted with oleylamine as a solvent or reagent, whereby tris(oleylamino)phosphine is rapidly produced *in situ* by transamination, followed by the reaction with indium halides.^33,34^ We find that the resulting InP NCs still show broader optical features compared to (TMS)_3_P-based preparations, unless a postsynthetic size-selective fractionation is applied.^30,32^ The quest for novel precursor chemistries for InP NCs is apparently not ceasing.

**Figure 1:**
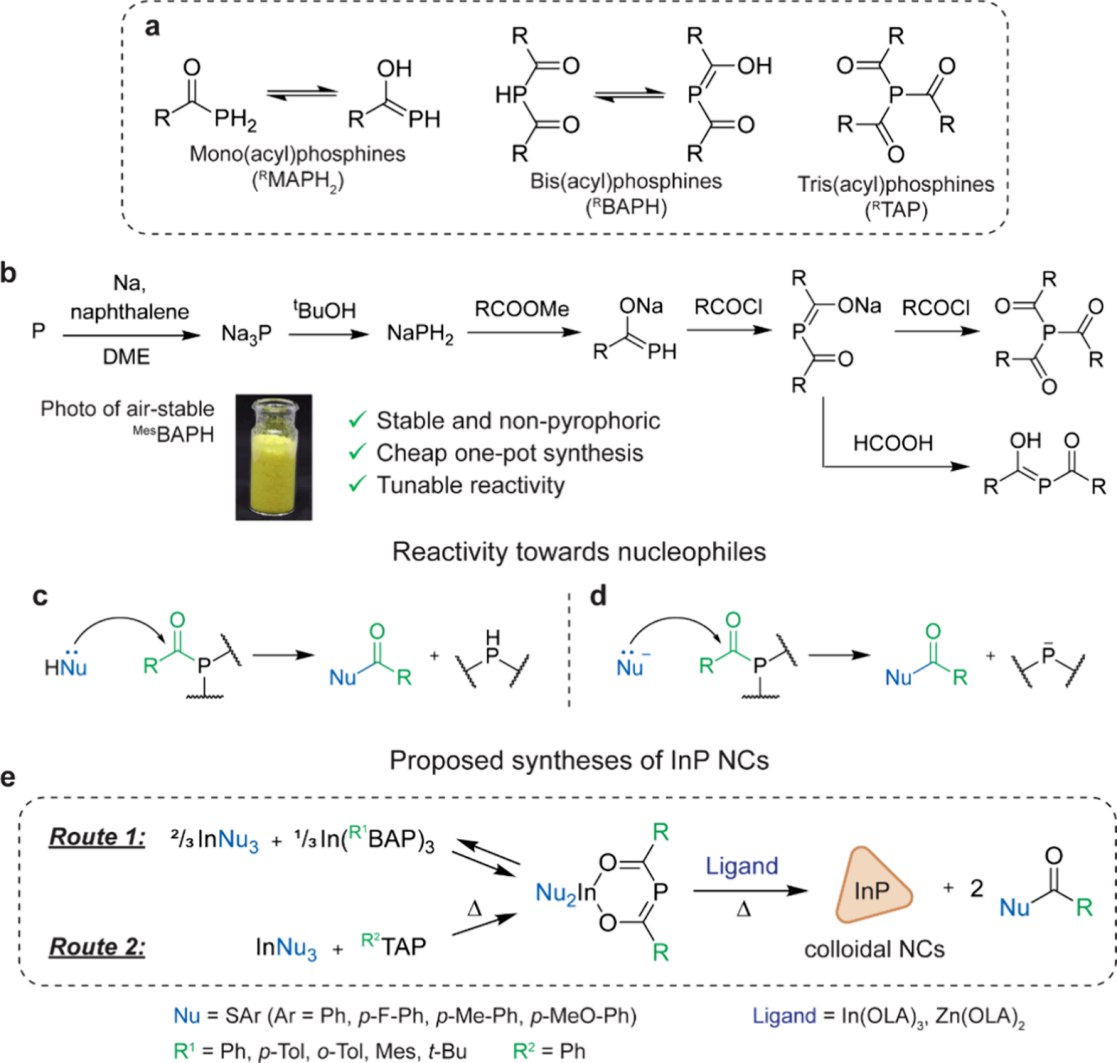
Acylphosphine precursors for the synthesis of InP QDs. (a) General structural formulas of mono-, bis-, and tris(acyl)phosphines. (b) Convenient one-pot synthesis of bis- and tris(acyl)phosphines from red phosphorus. (c, d) Reactivity of acylphosphines toward nucleophiles. (e) Proposed use of acylphosphines in the synthesis of colloidal InP NCs.

Here, we propose nonpyrophoric acylphosphines (Figure 1) as convenient phosphorus precursors for the synthesis of InP NCs. Bis(acyl)phosphines (BAPs) and tris(acyl)phosphines (TAPs), except for the simplest bis(acetyl)- and tris(acetyl)phosphines (^Me^BAPH and ^Me^TAP), are stable and nonvolatile solids, which either slowly degrade when stored in contact with air or are completely air-stable. Facile and low-cost syntheses of these compounds from metallic sodium and red phosphorus (Figure 1b),^35,36^ white phosphorus,^37^ or from PH_3_^38^ have been reported. Acylphosphines are then typically oxidized, as the corresponding acylphosphine oxides represent an important class of photoinitiators that are used in dentistry, for the fabrication of polymer coatings and in 3D-printing.^36,39,40^ We now demonstrate that TAPs and indium complexes of BAP anions can also be used as a formal source of the P^3–^ trianion in the synthesis of InP NCs, when combined with suitable anionic nucleophiles, such as alkoxides or arylthiolates (Figure 1e). This new synthetic protocol is utilized to synthesize uniform colloidal InP QDs with well-defined excitonic features across the visible region of the electromagnetic spectrum. Moreover, the reactivity of acylphosphines and thus the final size of the NCs (2–4 nm) can be adjusted by varying the acyl substituents or by replacing indium oleate ligand (In(OLA)_3_) with zinc oleate (Zn(OLA)_2_). We believe that such controllable precursor reactivity not only allows tuning of the NCs size at close to full reaction conversion, but may potentially open a gateway to InP NCs of anisotropic shapes and the related heteronanostructures. Future work shall also extend to other technologically important metal phosphides and arsenides, such as GaP, InAs, InGaAs and Fe_*x*_P.

## Experimental Section

The list of chemicals, the syntheses of acylphosphines, indium tris[bis(trimethylsilyl)amide] (In[N(TMS)_2_]_3_) and other InNu_3_ (Nu = nucleophile) compounds are available in the Supporting Information. All manipulations were carried out in a glovebox or by using standard Schlenk techniques.

### Syntheses of Complexes **1**–**5**

#### In(^Ph^BAP)_3_ (**1**)

In[N(TMS)_2_]_3_ (2.50 g, 4.2 mmol) was dissolved in tetrahydrofuran (THF, 20 mL) inside of a 250 mL two-neck flask connected to a Schlenk line. ^Ph^BAPH (2.90 g, 12 mmol) in THF (12 mL) was added dropwise and the reaction mixture was stirred for 1 h. The bright red crystalline product was precipitated by adding 100 mL of *n*-hexane (crystallization occurs over several hours), collected by filtration through a Schlenk frit, washed twice with 20 mL of *n*-hexane and dried under vacuum for 4 h. Yield – 2.77 g (83%). ^1^H NMR (C_6_D_6_, 300 MHz): δ = 8.34 (d, 12H, *o*-H), 7.00 (t, 6H, *p*-H), 6.90 (t, 12H, *m*-H) ppm. ^31^P NMR (C_6_D_6_, 121.5 MHz): δ = 73.5 (s) ppm.

#### In(^*p*-Tol^BAP)_3_ (**2**)

In[N(TMS)_2_]_3_ (1.25 g, 2.1 mmol) was dissolved in THF (10 mL) inside of a 250 mL two-neck flask connected to a Schlenk line. ^*p*-Tol^BAPH (1.62 g, 6 mmol) in THF (40 mL) was added dropwise and the reaction mixture was stirred for 1 h. The solution was evaporated under vacuum until the onset of crystallization, and the bright orange crystalline product was further precipitated by adding 80 mL of *n*-hexane. The product was collected by filtration through a Schlenk frit, washed twice with 10 mL of *n*-hexane and dried under vacuum for 4 h. Yield – 1.35 g (73%). ^1^H NMR (C_6_D_6_, 300 MHz): δ = 8.38 (d, 12H, *o*-H), 6.74 (d, 12H, *m*-H), 1.86 (s, 18H, CH_3_) ppm. ^31^P NMR (C_6_D_6_, 121.5 MHz): δ = 71.2 (s) ppm.

#### In(^*o*-Tol^BAP)_3_ (**3**)

In[N(TMS)_2_]_3_ (2.50 g, 4.2 mmol) was dissolved in THF (20 mL) inside of a 250 mL two-neck flask connected to a Schlenk line. ^*o*-Tol^BAPH (3.24 g, 12 mmol) in THF (12 mL) was added dropwise and the reaction mixture was stirred for 1 h. The solution was evaporated under vacuum until the onset of crystallization and the bright orange crystalline product was further precipitated by adding 75 mL of *n*-hexane and cooling the suspension to 0 °C. The product was collected by filtration through a Schlenk frit, washed twice with 10 mL of *n*-hexane and dried under vacuum for 4 h. Yield – 2.56 g (69%). ^1^H NMR (C_6_D_6_, 300 MHz): δ = 7.98 (d, 6H, *o*-H), 6.98–6.80 (m, 18H, *m*-H and *p*-H), 2.56 (s, 18H, CH_3_) ppm. ^31^P NMR (C_6_D_6_, 121.5 MHz): δ = 84.2 (s) ppm.

#### In(^*t*-Bu^BAP)_3_ (**4**)

In[N(TMS)_2_]_3_ (1.25 g, 2.1 mmol) was dissolved in THF (10 mL) inside of a 40 mL vial. ^*t*-Bu^BAPH (1.21 g, 6 mmol) in THF (10 mL) was added dropwise and the reaction mixture was stirred for 1 h. The solution was evaporated under vacuum and the residual was dissolved in a minimum amount of diethyl ether (5 mL). The solution was filtered through a PTFE filter and stored in a freezer (−35 °C) overnight. Yellow block crystals were collected by decanting out the mother liquor, washed with 1 mL of cold diethyl ether and dried under vacuum for 2 h. Additional fraction of the product was isolated by evaporating the mother liquor to half of the original volume and placing it into the freezer. Combined yield – 0.84 g (58%). ^1^H NMR (C_6_D_6_, 300 MHz): δ = 1.29 (d, 54H, *t*-Bu) ppm. ^31^P NMR (C_6_D_6_, 121.5 MHz): δ = 62.2 (s) ppm.

#### In(^Mes^BAP)_3_ (**5**)

In[N(TMS)_2_]_3_ (1.25 g, 2.1 mmol) was dissolved in THF (7 mL) inside of a 100 mL two-neck flask connected to a Schlenk line. ^Mes^BAPH (1.96 g, 6 mmol) in THF (6 mL) was added dropwise and the reaction mixture was stirred for 1 h. The bright yellow crystalline product was precipitated by adding 40 mL of *n*-hexane (crystallization takes at least 1 day), collected by filtration through a Schlenk frit, washed twice with 5 mL of *n*-hexane and dried under vacuum for 2 h. Yield – 1.50 g (69%). ^1^H NMR (C_6_D_6_, 300 MHz): δ = 6.60 (s, 12H, *m*-H), 2.47 (s, 36H, *o*-CH_3_), 2.02 (s, 18H, *p*-CH_3_) ppm. ^31^P NMR (C_6_D_6_, 121.5 MHz): δ = 96.5 (s) ppm.

### Solvothermal Experiments in High-Boiling-Point Solvents

Pure homoleptic In(^R^BAP)_3_ (0.04 mmol), heteroleptic In(Nu)_2_(^R^BAP) (0.04 mmol), or a mixture of In(^R^BAP)_3_ (0.0133 mmol) and InNu_3_ (0.0267 mmol) was loaded into a 4 mL vial together with 1 mL of 1-octadecene (ODE) or Diphyl (eutectic mixture of diphenyl and diphenyl ether) solvent and sealed with a PTFE-lined phenolic screw cap inside of a glovebox. While stirring, the temperature was increased stepwise with 25 °C intervals (10 min at each set point), and the progress of the reaction was visually monitored. If any solid residual was obtained after heating to 300 °C (in ODE) or 275 °C (in Diphyl), it was further precipitated by adding 1 mL of acetone, collected by centrifugation, washed once again with 1 mL of acetone and characterized with powder X-ray diffraction (XRD).

### Solvothermal Experiments in Toluene-d8

***WARNING! Teflon-lined autoclaves can be routinely operated at temperatures up to 200 °C and should never be operated above 250 °C or beyond their maximum pressure range. Danger of explosion!***

Pure homoleptic In(^R^BAP)_3_ (0.12 mmol), heteroleptic In(Nu)_2_(^R^BAP) (0.12 mmol), or a mixture of In(^R^BAP)_3_ (0.04 mmol) and InNu_3_ (0.08 mmol) was loaded into a 25 mL Teflon-lined autoclave together with 3 mL of toluene-d8, sealed inside of a glovebox and placed into a preheated muffle furnace. After 1 h at a target temperature, the autoclave was taken out from the furnace, cooled to room temperature and transferred into a glovebox. An aliquot (0.5 mL) was taken for NMR measurements and the procedure was repeated (up to two times) with heating to higher target temperatures.

### Preparation of In(OLA)_3_ and Zn(OLA)_2_ Stock Solutions

Indium acetate (1.87 g, 6.4 mmol), oleic acid (OLA, 6.1 mL, 19.2 mmol) and ODE (32 mL) were loaded into a 100 mL three-neck flask equipped with a thermocouple and connected to a Schlenk line through a reflux condenser. The atmosphere in the flask was exchanged by three cycles of consecutive switching from vacuum to argon gas. The reaction mixture was then heated to 120 °C under inert atmosphere and kept at this temperature for 10 min. Acetic acid that is produced in the reaction was carefully removed by applying vacuum. After this, the almost clear solution was additionally degassed at 120 °C overnight to remove any traces of the acetic acid. Finally, three additional vacuum/argon cycles were performed, the heating was turned off and the warm solution was transferred into a sealed nitrogen-filled vial using cannula technique.

Zn(OLA)_2_ stock solution was prepared following the same steps, except zinc acetate (1.17 g, 6.4 mmol) and OLA (4.0 mL, 12.8 mmol) were used. In(OLA)_3_ and Zn(OLA)_2_ stock solutions were stored inside of a nitrogen-filled glovebox. In case of phase separation, the solutions were heated to 100 °C shortly before use.

### Heating-Up Syntheses of InP QDs Following **Route 1**

Indium arylthiolate (In(SAr)_3_, 0.16 mmol) was loaded into a 25 mL three-neck flask equipped with a thermocouple and connected to a Schlenk line through a reflux condenser. In(OLA)_3_ (or Zn(OLA)_2_) stock solution in ODE (1.2 mL, 0.2 M) was added into the flask and further diluted with dry degassed ODE to a total volume of 6 mL. The reaction mixture was then heated to 150 °C (Ar = Ph) or 180 °C (Ar = *p*-F-Ph, *p*-MeO-Ph) under inert atmosphere and kept at this temperature for 10 min, leading to the complete dissolution of In(SAr)_3_. The reaction temperature was then decreased to 100 °C and a solution of In(^Ph^BAP)_3_ (67.0 mg, 0.08 mmol) in Diphyl (2 mL) was injected. The reaction mixture was then rapidly heated to 275 °C and kept at this temperature for 1 h. Progress of the reaction was monitored by taking aliquots and quenching them in dry degassed toluene. The reaction was stopped by rapidly cooling the flask to room temperature and the contents of the flask were transferred into a glovebox to purify the NCs.

### Hot-Injection Syntheses of InP QDs Following **Route 1** (See the Supporting Information for **Route 2**)

An empty 25 mL three-neck flask was equipped with a thermocouple and a rubber septum and connected to a Schlenk line through a reflux condenser. The atmosphere in the flask was exchanged by three cycles of consecutive switching from vacuum to argon gas. In(OLA)_3_ (or Zn(OLA)_2_) stock solution in ODE (1.2 mL, 0.2 M) was added into the flask and further diluted with dry degassed ODE to a total volume of 6 mL. The flask was then heated to 285 °C. In parallel, an injection solution was prepared by dissolving In(^R^BAP)_3_ (0.08 mmol) and In(SAr)_3_ (0.16 mmol) in Diphyl (1 mL) at 125 °C (R = Ph, *p*-Tol, *o*-Tol, *t*-Bu) or 175 °C (R = Mes) for 30 min, leading to the formation of wine-red indium mono(acyl)phosphide (In-MAP) intermediate. The obtained solution was quickly injected into the reaction flask, and the thermocontroller was set at 275 °C. After a certain time (Table S1), the reaction was quenched by rapid cooling to room temperature, and the contents were transferred into a glovebox to purify the NCs.

### Purification of the NCs

The NCs were precipitated by adding excess acetone (up to 30 mL, depending on the synthesis) or a mixture of acetone (20 mL) and ethanol (up to 20 mL) and collected by centrifugation (19’800 RCF, 5 min). The obtained precipitate was redispersed in 2 mL of *n*-hexane. Another purification cycle was performed using acetone or ethanol as a bad solvent. Finally, the obtained dispersion of InP NCs in *n*-hexane was filtered through a 0.2 μm PTFE filter and stored in the glovebox. Typical yields of the isolated InP NCs – 40–80% (based on optically determined InP concentration).^41^

## Results and Discussion

### Synthesis and Chemical Reactivity of Acylphosphines

Different BAPs and TAPs were synthesized according to a previously reported procedure (Figure 1b),^35,36,42^ wherein metallic sodium and red phosphorus are first reacted in the presence of naphthalene as electron transfer promotor to produce sodium phosphide, which is then solubilized by partial protonation with *tert-*butanol and sequentially acylated to produce sodium salts of BAPs and TAPs, respectively. All synthetic steps take place in one flask, allowing convenient production of diverse acylphosphines on a multigram scale with good isolated yields (40–90%).

Acylphosphines are known to be reactive toward nucleophiles (Figure 1c,d), which is typically considered their downside as it causes undesirable degradation in industrial applications exploiting their photohomolysis.^39^ In this work, we utilize this type of reactivity and demonstrate that acylphosphines can be used as safe and efficient sources of the P^3–^ trianion in the synthesis of metal phosphide NCs, namely InP QDs. Indeed, ^Ph^TAP readily reacts with oleylamine, a typical ligand in the synthesis of semiconductor NCs, leading to a stepwise elimination of the acyl groups in the form of N-oleylbenzamide and eventual formation of PH_3_ (Figure S1a,b). This type of ^Ph^TAP “activation” can be used in the synthesis of InP NCs, but it produces unsatisfactory results in terms of NCs size distribution (Figure S1c) due to a dynamic equilibrium between different protonated forms of the phosphide intermediates and the volatile nature of PH_3_.^43^ Interestingly, ^Ph^TAP also reacts with indium carboxylates, which are common indium precursors in the (TMS)_3_P-based syntheses, leading to the formation of In-BAP complexes and carboxylic anhydride as a byproduct (Figure S2). In this case, protonation of the bis(acyl)phosphide anion is not possible, and the observed heteroleptic In-BAP complexes can serve as viable intermediates in the synthesis of InP NCs. The reaction, however, does not proceed to completeness by increasing the temperature due to an insufficient driving force for the deacylation by carboxylates.

Inspired by the observed reactivity of ^Ph^TAP, we then conceived two possible synthetic routes to InP NCs from acylphosphines. Both of them rely on the intermediate formation of the heteroleptic In(Nu)_2_(^R^BAP) complexes that can decompose at high temperatures in the presence of long-chain ligands, eliminating two equivalents of the corresponding NuC(O)R byproducts (Figure 1e). On the first route, the intermediate heteroleptic In(Nu)_2_(^R^BAP) complexes are produced from the homoleptic ones (Figure 1e, **Route 1**), while the second route begins from the corresponding TAPs (Figure 1e, **Route 2**). The driving force and the kinetics of the deacylation reaction can be controlled by altering the nature of the nucleophile and substituents at the acyl groups. Since In-BAP complexes lie one reaction step closer to the target InP, we first explore **Route 1** by synthesizing a range of homoleptic In(^R^BAP)_3_ complexes and testing them in the reactions with different nucleophiles.

### Reactions of In(^R^BAP)_3_ Complexes with Various Molecular InNu_3_ Compounds

**Scheme 1:**
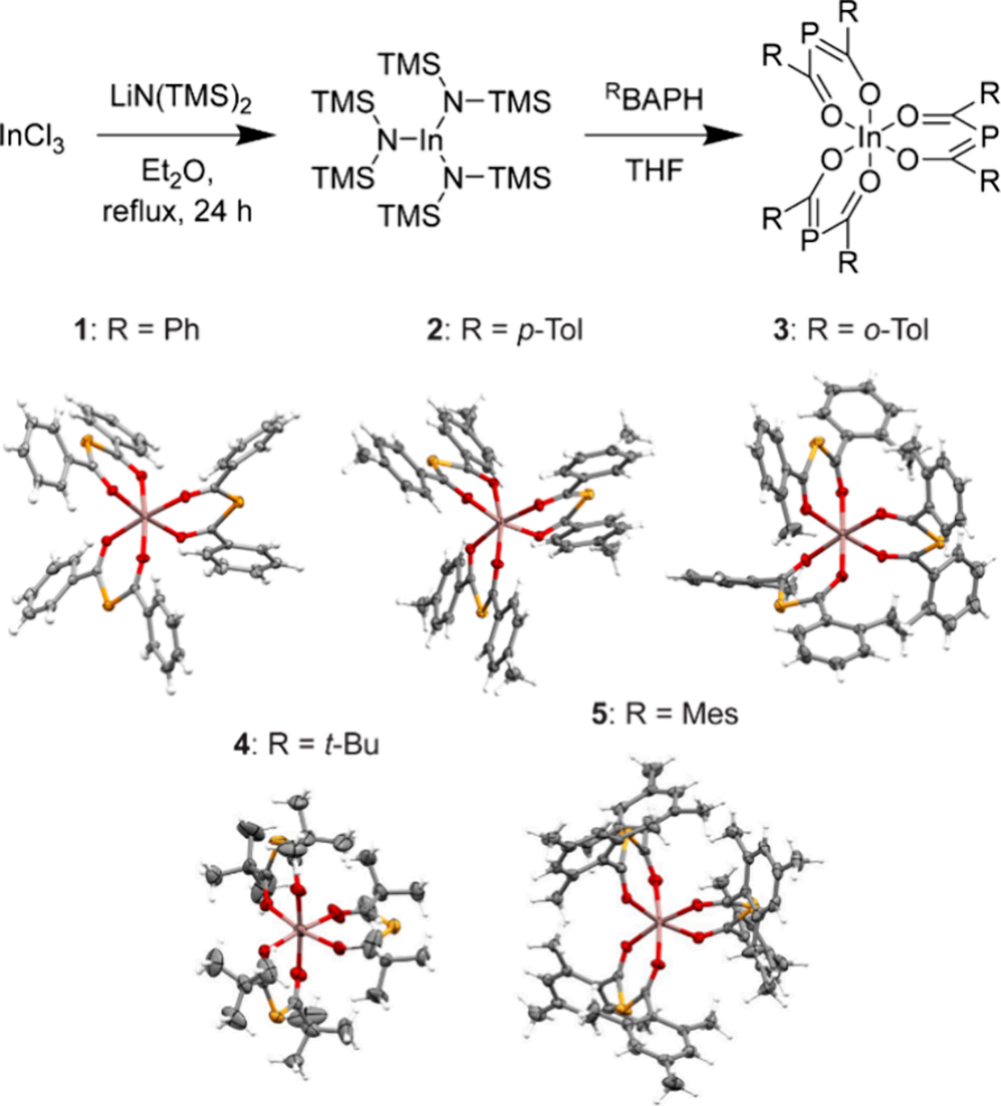
Synthesis and Molecular Structures of In(^R^BAP)_3_ Complexes in the Solid State

In(^R^BAP)_3_ complexes were synthesized by a ligand exchange reaction starting from In[N(TMS)_2_]_3_ (Scheme 1). Homoleptic complexes **1**–**5** were isolated in pure form as brightly colored crystalline solids (60–85% yields, see the Experimental Section for details). Crystal structures of the complexes **1–5** confirm that the ^R^BAP^–^ ligands are bound to the central In^3+^ via both oxygen atoms, in line with previous reports of BAP complexes with hard Lewis acids, such as Al^3+^,^42,44^ Ti^3+^,^35^ U^4+^, U^3+^ and Y^3+^,^45^ Eu^3+^ and Eu^2+^.^46,47^ Notably, metal coordination by the phosphorus center has also been reported; some examples include BAP complexes with group VIII metals, such as Ni^2+^,^48^ Fe^2+^,^49^ Os^2+^,^50^ and Ru^2+^.^51^ Despite the absence of In–P bonding in the In-BAP complexes, we hypothesized that the next deacylation step should lead to the formation of an In–P bond (see the discussion below), opening a potential pathway to InP.

**Chart 1:**
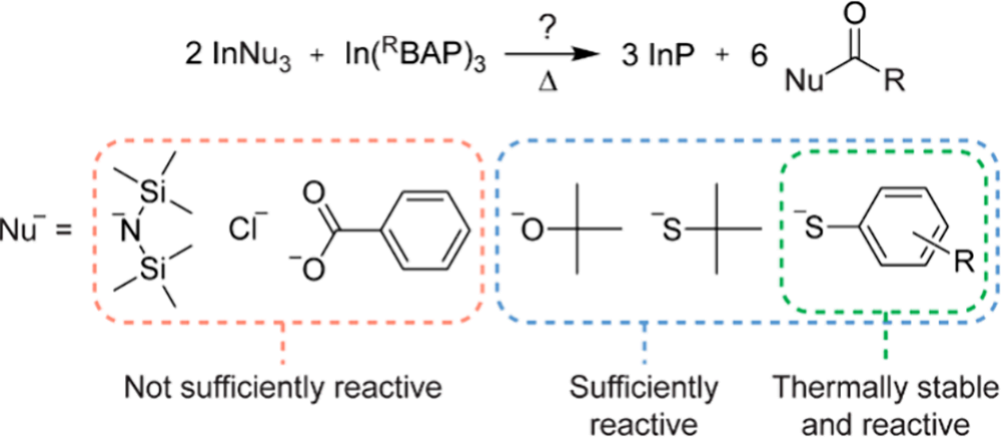
Various Nucleophile Anions Ranked According to Their Ability to Form InP by the Removal of Acyl Groups from In-BAP Complexes and TAPs

**Figure 2:**
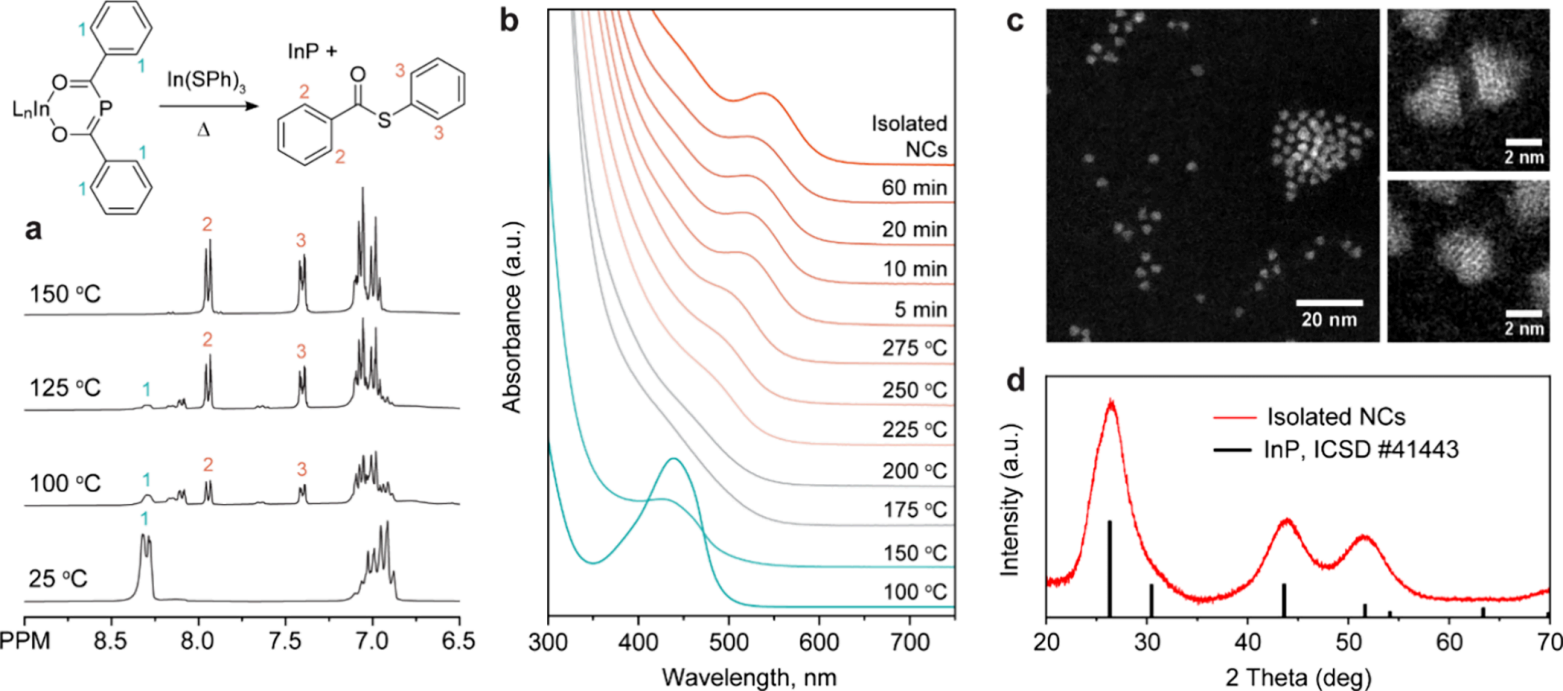
(a) Reaction of In(^Ph^BAP)_3_ with In(SPh)_3_ in toluene-d8 studied with ^1^H NMR. Formation of the thioester byproduct **10b** is evidenced by the gradual appearance of doublets 2 and 3. (b) Heating-up synthesis of InP NCs from In(^Ph^BAP)_3_ and In(SPh)_3_ in the presence of In(OLA)_3_. The ^Ph^BAP ligand in the starting material (strong absorption band around 440 nm) gradually vanishes at 100–150 °C, whereas nucleation and eventual growth of InP NCs are observed above 225 °C. (c) HAADF-STEM and (d) powder XRD of the isolated NCs. Background in the XRD pattern was subtracted using rubberband baseline construction.

To test this reasoning, we opted for molecular InNu_3_ compounds (Chart 1, Figure S3) as both the well-defined deacylation reagents and the In^3+^ sources, permitting understanding of the reaction mechanism. Various InNu_3_ complexes were synthesized and tested in the reactions with complexes **1** and **5** that differ by the amount of steric hindrance at the acyl groups. The experiments were performed with a stoichiometric InNu_3_:In(^R^BAP)_3_ ratio of 2:1 in deuterated toluene inside an autoclave, with gradually increasing reaction temperature (see the Experimental Section for more details). The progress of the reactions was monitored with ^1^H and ^31^P NMR. Mild heating of the reaction mixtures led to the expected redistribution of ligands with the formation of equilibrium mixtures of heteroleptic and homoleptic complexes. In some cases, the corresponding heteroleptic complexes **6**-**8** could be isolated in pure form and the crystal structures were determined (Figure S4). Further reaction pathways, however, differ depending on the nature of the nucleophile anion (results are briefly summarized in Chart 1). While chloride and benzoate are capable of reversibly removing one acyl group from ^Ph^TAP (Figure S5), further deacylation seems to be uphill in energy and is not observed. Instead, In-BAP complexes decompose at ca. 150 °C (R = Ph) and ca. 250 °C (R = Mes), leaving the starting InCl_3_ and indium benzoate intact (Figures S6–S9). Similarly, the bis(trimethylsilyl)amide anion was also found to be insufficiently reactive due to its high steric hindrance and low nucleophilicity (Figures S10 and S11). Indium alkoxides and thiolates, on the other hand, are more nucleophilic, but their aliphatic versions lack sufficient thermal stability. While indium *tert*-butoxide, [In(O^t^Bu)_3_]_2_, successfully reacts with In(^Ph^BAP)_3_ above 100 °C, forming the corresponding ester **9** and nanocrystalline InP (Figure S12), it decomposes to indium oxide, isobutylene and *tert*-butanol when heated together with In(^Mes^BAP)_3_ which requires higher temperatures for the deacylation reaction (Figure S13). Indium *tert*-butylthiolate, [In(S^t^Bu)_3_]_2_, was found to decompose even at lower temperatures when heated with both complexes **1** and **5** (Figure S14). Proper combination of thermal stability and sufficient reactivity was found for indium arylthiolates, In(SAr)_3_. Indium thiophenolate, In(SPh)_3_, reacts with complexes **1** and **5** above 100 and 200 °C, respectively, resulting in the elimination of the thioesters **10b** and **11b** and the formation of nanocrystalline InP (Figure 2a, Figure S15).

Indium arylthiolates can be conveniently synthesized in pure form (i.e., described by a defined molecular formula) in a single step from indium metal and the corresponding aryldisulfides,^52^ despite their polymeric nature that is confirmed here with the crystal structure of In(SPh-F-*p*)_3_ (Figure S16). Chemical bonds in the polymer chains of indium arylthiolates are sufficiently labile to enable good solubility in hot nonpolar aromatic solvents, making them suitable reagents for the synthesis of colloidal InP NCs. Furthermore, arylthiolates can be functionalized with various substituents at the phenyl rings, allowing precise control of their reactivity and solubility in aliphatic and aromatic solvents.

### Colloidal Synthesis of InP NCs

The outlined chemistry was then adopted for the colloidal synthesis of InP QDs by performing the same reactions in the presence of “chemically inert” (i.e., not reactive toward BAPs) long-chain metal (In^3+^ or Zn^2+^) carboxylate ligands. In a typical heating-up synthesis, In(SPh)_3_ is first dissolved in a solution of In(OLA)_3_ in ODE at 150 °C, after which the temperature is decreased to 100 °C and a solution of In-BAP complex **1** in Diphyl is injected. The temperature is then increased to 275 °C and the progress of the reaction is monitored by taking aliquots and quenching them in dry degassed toluene. Absorption spectra of the reaction aliquots reveal rapid conversion of the starting ^Ph^BAP ligand between 100 and 150 °C followed by the nucleation of InP NCs above 225 °C (Figure 2b). The excitonic peak sharpens and red-shifts until the NCs reach their final size after ca. 10–20 min of the reaction at 275 °C. High-angle annular dark-field scanning transmission electron microscopy (HAADF-STEM) reveals relatively monodisperse NCs of a rounded tetrahedral shape, with an average size of 3.0 nm and a standard deviation of 13% (Figure 2c). The crystalline nature of the obtained InP NCs is evident from powder XRD (Figure 2d, Figures S17–S20) and high-resolution STEM images (Figure 2c). The sufficient thermal stability of the arylthiolate precursors is further highlighted by the absence of any detectable sulfur contamination in the purified samples, as attested by the results of energy-dispersive X-ray (EDX) spectroscopy (Figure S21).

**Figure 3:**
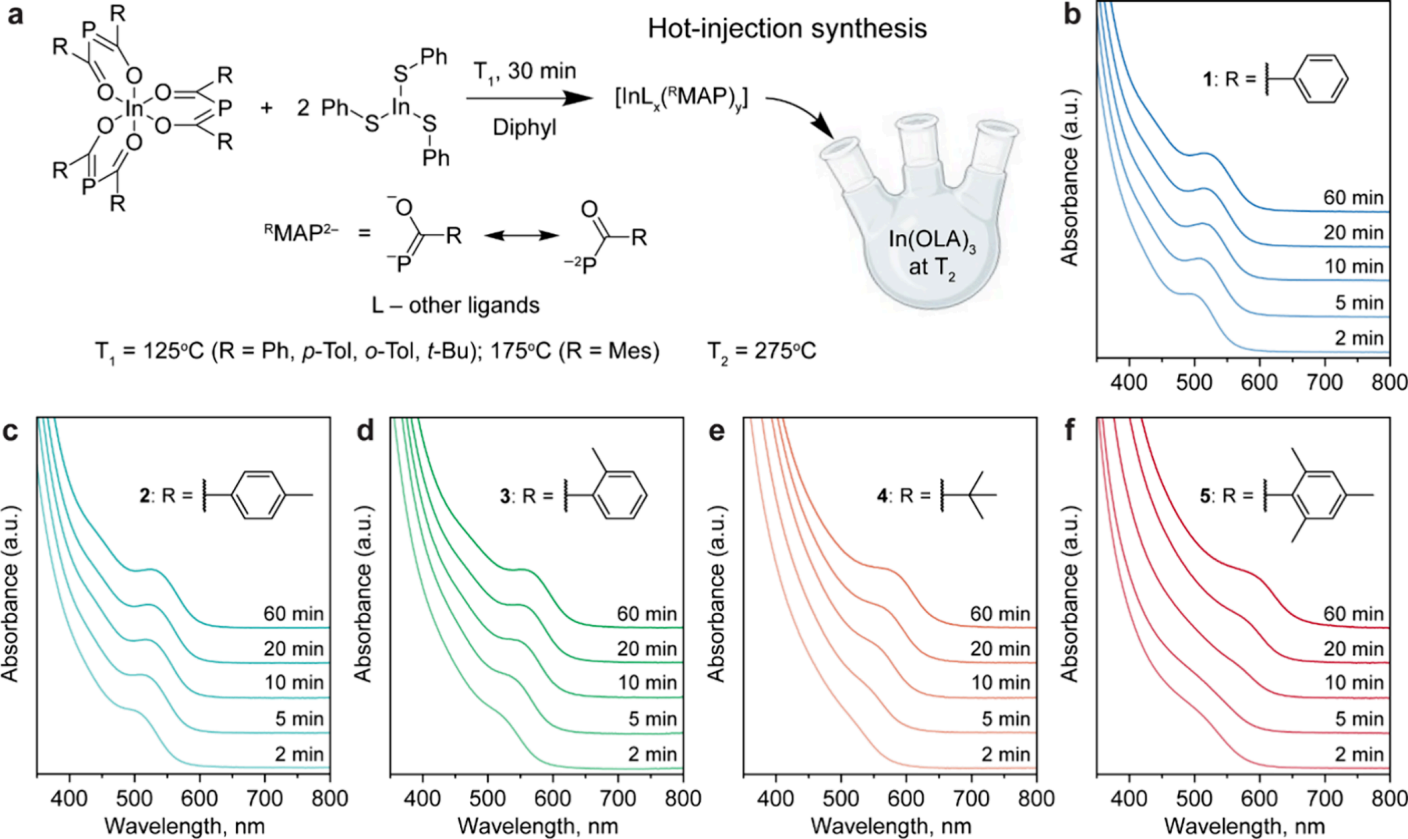
Influence of acyl substituents on the kinetics of InP formation in the hot-injection syntheses at 275 °C. (a) Schematic of the hot-injection syntheses. Injection solutions are prepared by dissolving complexes **1**–**5** together with In(SPh)_3_ in hot Diphyl, thereby forming the presumed In-^R^MAP intermediates. (b–f) Temporal evolution of the absorption spectra in the syntheses of InP NCs from complexes **1** (b), **2** (c), **3** (d), **4** (e), and **5** (f). Bulkier substituents lead to slower InP nucleation and growth under otherwise equal reaction conditions.

Note that the optical absorption spectra of the reaction aliquots taken between 175 and 200 °C (Figure 2b) are devoid of any distinct features and appear rather similar independent of the actual synthesis procedure (Figure S22). We hypothesize that the absorptions may correspond to an intermediate ^Ph^MAP^2–^ dianion that is formed by removal of one acyl group from the ^Ph^BAP^–^ ligand (Figure 3a). The ^Ph^MAP^2–^ anion is expected to strongly bind to one or multiple In^3+^ cations in a multidentate fashion involving both the phosphorus and the oxygen atoms. We were not able to isolate such an [InL_*x*_(^Ph^MAP)_*y*_] (L – other ligands, e.g. arylthiolate, carboxylate, etc.) intermediate in pure form likely due to its polymeric nature in the solid state, but acidification of the intermediate solution in Diphyl with citric acid leads to the release of ^Ph^MAPH_2_ along with PH_3_ (Figure S23). Formation of the latter can be explained by the reaction of ^Ph^MAPH_2_ with a simultaneously liberated thiophenol. The [InL_*x*_(^R^MAP)_*y*_] (in short, In-^R^MAP) intermediates can be also prepared separately as solutions in Diphyl and used in hot-injection syntheses of InP NCs by injecting them into a preheated solution of the long-chain metal carboxylate ligand (Figure 3a, more details in the Experimental Section). We will focus our further discussion on the hot-injection method, as it produces QD samples with slightly better defined excitonic features as compared to the heating-up method (Figure S25).

**Figure 4:**
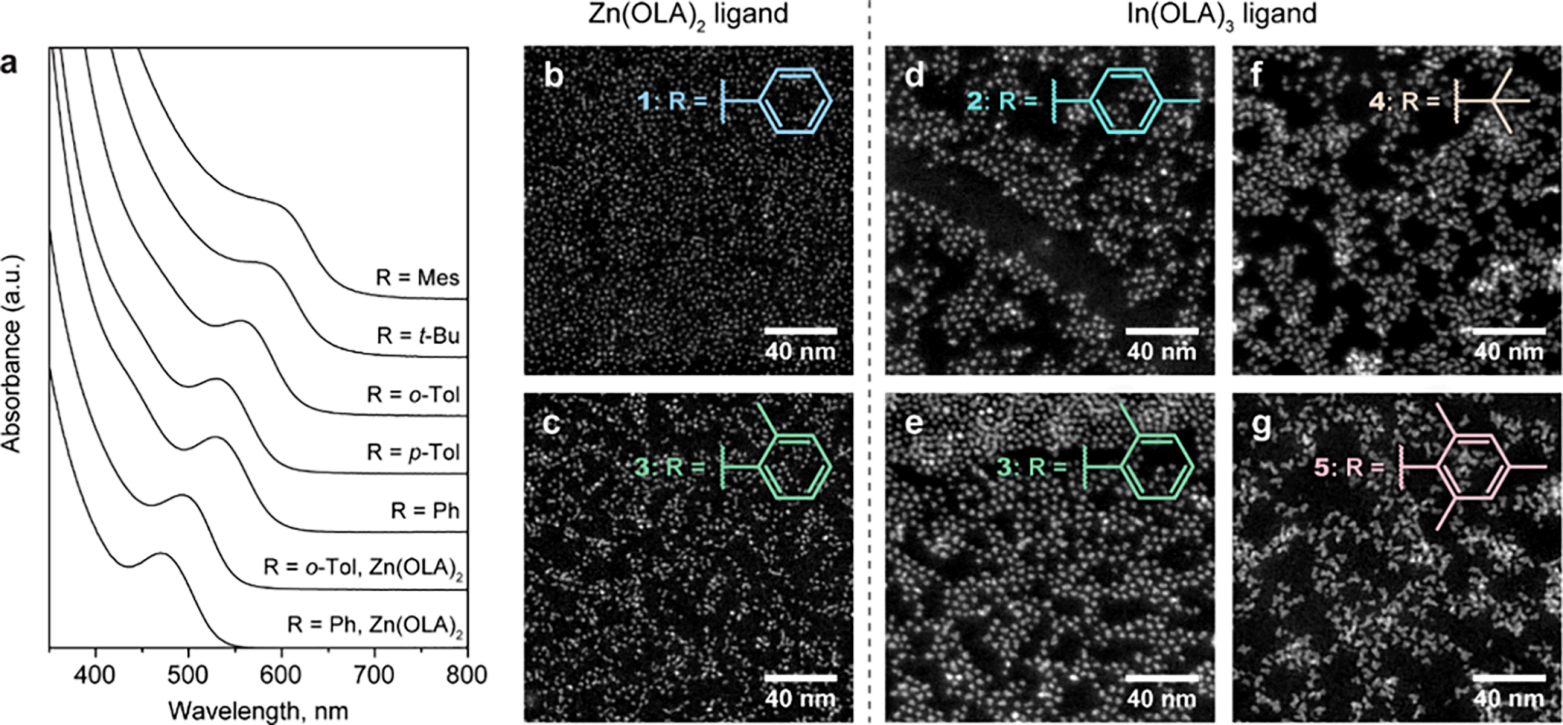
Control of the average NC size by varying the acyl substituents and the metal carboxylate ligand. (a–g) Absorption spectra (a) and HAADF-STEM images (b–g) of InP NCs that were synthesized from **1** in the presence of Zn(OLA)_2_ (b), **3** in the presence of Zn(OLA)_2_ (c), **2** in the presence of In(OLA)_3_ (d), **3** in the presence of In(OLA)_3_ (e), **4** in the presence of In(OLA)_3_ (f), and **5** in the presence of In(OLA)_3_ ligand (g). Average NC sizes are 2.1, 2.4, 2.8, 3.3, 3.6, and ≈4 nm, respectively.

The ability to control conversion kinetics, and therefore the average NC size at full reaction conversion, is important for minimizing batch-to-batch variability at industrially relevant reaction scales.^10^ Therefore, we explored the possibility of adjusting the conversion kinetics in the proposed synthesis by changing substituents in the aryl rings of indium arylthiolates and at the acyl groups of BAPs. It was found that the presence of electron donating (Me-, MeO-) and electron withdrawing (F-) groups in the *para*- position of the arylthiolate has a minor effect both on the kinetics of the first reaction step (Figures S26 and S27) and on the size of the resulting NCs (Figure S28). The bulkiness of substituents in the ^R^BAP moiety, instead, strongly influences the rates of both reaction steps and, as a result, it also affects the final size of the resulting NCs. Indeed, sterically hindered complex **5** (R = Mes) starts to react with indium arylthiolates at a temperature, which is ca. 100 °C higher compared to the sterically more accessible complex **1** (R = Ph) (Figures S26 and S27). The rate of InP NCs formation at 275 °C follows a similar trend and occurs much slower for complex **5** than **1** under otherwise equal conditions (Figure 3b,f). Similarly, compounds **2**, **3** and **4** with moderate degrees of steric hindrance convert into InP at time scales that are intermediate between those of complexes **1** and **5** (Figure 3c–e). As a result, the excitonic peak position of the final InP QDs can be tuned between 530 and 600 nm (Figure 4a), corresponding to the change of NCs size from 2.8 to 4 nm (Figure 4d–g). Even smaller NCs (down to 2.1 nm and less) can be synthesized by replacing In(OLA)_3_ ligand with Zn(OLA)_2_ (Figure 4a–c), which is known to favor nucleation of small InP QDs.^53^ Note that the NCs larger than 3.5 nm adopt an irregular morphology (Figure 4f,g), which translates into the smearing of their excitonic features in the absorption spectra. This morphological change may be attributed to side reactions competing with the growth of InP in the case of slowly reacting acylphosphines, or to suboptimal reaction conditions. Overall, altering the chemical structure of acylphosphines does tune the conversion kinetics and therefore, to some extent, also the final NCs size at full reaction conversion. Besides the steric effects, changing the electronic properties of acylphosphines represents another way of tuning their reactivity and, therefore, will be explored in our future studies.

Extension of the proposed chemistry to other III–V materials and transition metal phosphides might benefit from eliminating the need to synthesize the mixed metal-pnictogen precursors. This is feasible on **Route 2**, starting from the corresponding TAPs (Figure 1e), which can be potentially combined with any suitable metal-nucleophile (MNu_*x*_) precursor. Here, too, we chose the hot-injection approach, wherein ^Ph^TAP is first reacted with In(SPh)_3_ to form the assumed In-^Ph^MAP intermediate, which is then injected into a hot solution of the corresponding metal oleate (see the Supporting Information for details). Despite a higher amount of the carboxylate ligand that was required to produce colloidally stable dispersions, the syntheses according to **Route 2** yielded InP NCs of similar quality (average size, morphology and uniformity) as with **Route 1** (Figures S29 and S30), attesting it as a potent path to metal phosphide NCs. The need to use higher amounts of oleate ligands in **Route 2** suggests their participation and gradual consumption during the NCs synthesis. While carboxylates are unlikely to be capable of deacylating the In-^Ph^MAP intermediate, they might slowly undergo a reversible transesterification with the thioester byproduct **10b**, leading to a gradual replacement of surface-bound oleate with short and rigid benzoate that does not support good colloidal stability. In the case of complexes **4** and **5**, bulky pivalate and 2,4,6-trimethylbenzoate formed in this way may even block the surface of InP NCs, therefore hindering their further growth and leading to the observed irregular polycrystalline morphology. To this end, the future work shall explore the use of long-chain arylthiolates and aryloxides as both the nucleophilic reagents and the ligands for the syntheses of metal phosphide and metal arsenide NCs according to the proposed acylpnictide route.

## Conclusions

In summary, we have explored diverse solid, nonpyrophoric, and synthetically readily accessible acylphosphines as potent precursors to InP NCs. Leveraging their reactivity toward various nucleophiles, we proposed and experimentally validated two convenient synthetic routes to InP QDs. The first route relies on using air-stable In(^R^BAP)_3_ complexes as mixed phosphorus–indium precursors, whereas the second route begins with TAPs. Both the In(^R^BAP)_3_ complexes and the TAPs can be sequentially deacylated (with In-MAPs as the final detectable intermediates) by suitable anionic nucleophiles in the presence of long-chain metal carboxylates, ultimately leading to colloidal InP NCs. The optimal choice of nucleophiles and acylphosphines is determined by a balance between their reactivity and thermal stability. In particular, arylthiolates appear as suitable nucleophiles for a range of acylphosphines with different reactivities. Adjusting the size of acyl groups in the acylphosphine, instead, provides control over the conversion kinetics and, therefore, the final size of the resulting InP NCs. Further control of the NCs size is achieved by introducing Zn(OLA)_2_ as a ligand, affording synthesis of small 2 nm InP QDs that are relevant for green light emission. As synthesized InP QDs, however, show only weak photoluminescence. Epitaxial shelling with wider bandgap materials^9,31^ or other surface treatments^54,55^ would be required to fully assess their practical potential. In general, the proposed syntheses of InP NCs are rather robust, i.e. insensitive to small variations in the reaction stoichiometry and temperature (Figure S31), and can be further optimized by, for instance, replacing long-chain carboxylates with long-chain arylthiolates that can simultaneously act as nucleophiles and as ligands for NCs. The discovered acylpnictide chemistry might also foster the exploration of other technologically important metal phosphides and arsenides, such as GaP, InAs, InGaAs and Fe_*x*_P, in the colloidal form.

## Supporting Information Available

Additional experimental details, reactions of ^Ph^TAP with oleylamine and indium myristate, reactions of complexes **1** and **5** with various molecular InNu_3_ compounds, characterization of In-MAP intermediates, additional details of the colloidal InP NC syntheses, and crystallographic data (PDF)

## References

[ref1] Almeida G.; Ubbink R. F.; Stam M.; du Fossé I.; Houtepen A. J. InP Colloidal Quantum Dots for Visible and Near-Infrared Photonics. Nat. Rev. Mater. 2023, 8, 742–758. 10.1038/s41578-023-00596-4.

[ref2] Jang E.; Kim Y.; Won Y. H.; Jang H.; Choi S. M. Environmentally Friendly InP-Based Quantum Dots for Efficient Wide Color Gamut Displays. ACS Energy Lett. 2020, 5 (4), 1316–1327. 10.1021/acsenergylett.9b02851.

[ref3] Hahm D.; Ko D.; Jeong B. G.; Jeong S.; Lim J.; Bae W. K.; Lee C.; Char K. Environmentally Benign Nanocrystals: Challenges and Future Directions. J. Inf. Disp. 2019, 20 (2), 61–72. 10.1080/15980316.2019.1614487.

[ref4] Micic O. I.; Curtis C. J.; Jones K. M.; Sprague J. R.; Nozik A. J. Synthesis and Characterization of InP Quantum Dots. J. Phys. Chem. 1994, 98 (19), 4966–4969. 10.1021/j100070a004.

[ref5] Murray C. B.; Norris D. J.; Bawendi M. G. Synthesis and Characterization of Nearly Monodisperse CdE (E = Sulfur, Selenium, Tellurium) Semiconductor Nanocrystallites. J. Am. Chem. Soc. 1993, 115 (19), 8706–8715. 10.1021/ja00072a025.

[ref6] Sadasivan S.; Bausemer K.; Corliss S.; Pratt R. 27–1: Invited Paper: Performance Benchmarking of Wide Color Gamut Televisions and Monitors. SID Int. Symp. Dig. Tech. Pap. 2016, 47 (1), 333–335. 10.1002/sdtp.10672.

[ref7] Chen H.; He J.; Wu S. T. Recent Advances on Quantum-Dot-Enhanced Liquid-Crystal Displays. IEEE J. Sel. Top. Quantum Electron. 2017, 23 (5), 1–11. 10.1109/JSTQE.2017.2649466.

[ref8] Jo D. Y.; Kim H. M.; Park G. M.; Shin D.; Kim Y.; Kim Y. H.; Ryu C. W.; Yang H. Unity Quantum Yield of InP/ZnSe/ZnS Quantum Dots Enabled by Zn Halide-Derived Hybrid Shelling Approach. Soft Sci. 2024, 4, 27 10.20517/ss.2024.19.

[ref9] Won Y. H.; Cho O.; Kim T.; Chung D. Y.; Kim T.; Chung H.; Jang H.; Lee J.; Kim D.; Jang E. Highly Efficient and Stable InP/ZnSe/ZnS Quantum Dot Light-Emitting Diodes. Nature 2019, 575, 634–638. 10.1038/s41586-019-1771-5. 31776489

[ref10] Hendricks M. P.; Campos M. P.; Cleveland G. T.; Jen-La Plante I.; Owen J. S. A Tunable Library of Substituted Thiourea Precursors to Metal Sulfide Nanocrystals. Science 2015, 348 (6240), 1226–1230. 10.1126/science.aaa2951. 26068846

[ref11] Pun A. B.; Mazzotti S.; Mule A. S.; Norris D. J. Understanding Discrete Growth in Semiconductor Nanocrystals: Nanoplatelets and Magic-Sized Clusters. Acc. Chem. Res. 2021, 54 (7), 1545–1554. 10.1021/acs.accounts.0c00859. 33660971

[ref12] Li Y.; Hou X.; Dai X.; Yao Z.; Lv L.; Jin Y.; Peng X. Stoichiometry-Controlled InP-Based Quantum Dots: Synthesis, Photoluminescence, and Electroluminescence. J. Am. Chem. Soc. 2019, 141 (16), 6448–6452. 10.1021/jacs.8b12908. 30964282

[ref13] Xu Z. H.; Li Y.; Li J. Z.; Pu C. D.; Zhou J. H.; Lv L. L.; Peng X. G. Formation of Size-Tunable and Nearly Monodisperse InP Nanocrystals: Chemical Reactions and Controlled Synthesis. Chem. Mater. 2019, 31 (14), 5331–5341. 10.1021/acs.chemmater.9b02292.

[ref14] Ramasamy P.; Kim N.; Kang Y. S.; Ramirez O.; Lee J. S. Tunable, Bright, and Narrow-Band Luminescence from Colloidal Indium Phosphide Quantum Dots. Chem. Mater. 2017, 29 (16), 6893–6899. 10.1021/acs.chemmater.7b02204.

[ref15] Battaglia D.; Peng X. G. Formation of High Quality InP and InAs Nanocrystals in a Noncoordinating Solvent. Nano Lett. 2002, 2 (9), 1027–1030. 10.1021/nl025687v.

[ref16] Allen P. M.; Walker B. J.; Bawendi M. G. Mechanistic Insights into the Formation of InP Quantum Dots. Angew. Chem., Int. Ed. 2010, 49 (4), 760–762. 10.1002/anie.200905632. PMC3119352 20025010

[ref17] Koh S.; Eom T.; Kim W. D.; Lee K.; Lee D.; Lee Y. K.; Kim H.; Bae W. K.; Lee D. C. Zinc-Phosphorus Complex Working as an Atomic Valve for Colloidal Growth of Monodisperse Indium Phosphide Quantum Dots. Chem. Mater. 2017, 29 (15), 6346–6355. 10.1021/acs.chemmater.7b01648.

[ref18] Joung S.; Yoon S.; Han C. S.; Kim Y.; Jeong S. Facile Synthesis of Uniform Large-Sized InP Nanocrystal Quantum Dots using Tris(*tert*-butyldimethylsilyl)phosphine. Nanoscale Res. Lett. 2012, 7, 93 10.1186/1556-276X-7-93. 22289352 PMC3292828

[ref19] Franke D.; Harris D. K.; Xie L.; Jensen K. F.; Bawendi M. G. The Unexpected Influence of Precursor Conversion Rate in the Synthesis of III-V Quantum Dots. Angew. Chem., Int. Ed. 2015, 54 (48), 14299–14303. 10.1002/anie.201505972. 26437711

[ref20] Chandrasiri H. B.; Kim E. B.; Snee P. T. Sterically Encumbered Tris(trialkylsilyl) Phosphine Precursors for Quantum Dot Synthesis. Inorg. Chem. 2020, 59 (21), 15928–15935. 10.1021/acs.inorgchem.0c02440. 33040524

[ref21] Gary D. C.; Glassy B. A.; Cossairt B. M. Investigation of Indium Phosphide Quantum Dot Nucleation and Growth Utilizing Triarylsilylphosphine Precursors. Chem. Mater. 2014, 26 (4), 1734–1744. 10.1021/cm500102q.

[ref22] Harris D. K.; Bawendi M. G. Improved Precursor Chemistry for the Synthesis of III-V Quantum Dots. J. Am. Chem. Soc. 2012, 134 (50), 20211–20213. 10.1021/ja309863n. 23228014 PMC3535303

[ref23] Bang E.; Choi Y.; Cho J.; Suh Y. H.; Ban H. W.; Son J. S.; Park J. Large-Scale Synthesis of Highly Luminescent InP@ZnS Quantum Dots Using Elemental Phosphorus Precursor. Chem. Mater. 2017, 29 (10), 4236–4243. 10.1021/acs.chemmater.7b00254.

[ref24] Li L.; Protiere M.; Reiss P. Economic Synthesis of High Quality InP Nanocrystals Using Calcium Phosphide as the Phosphorus Precursor. Chem. Mater. 2008, 20 (8), 2621–2623. 10.1021/cm7035579.

[ref25] Liu Z.; Kumbhar A.; Xu D.; Zhang J.; Sun Z.; Fang J. Coreduction Colloidal Synthesis of III-V Nanocrystals: the Case of InP. Angew. Chem., Int. Ed. 2008, 47 (19), 3540–3542. 10.1002/anie.200800281. 18383567

[ref26] Yu P.; Shan Y.; Cao S.; Hu Y.; Li Q.; Zeng R.; Zou B.; Wang Y.; Zhao J. Inorganic Solid Phosphorus Precursor of Sodium Phosphaethynolate for Synthesis of Highly Luminescent InP-Based Quantum Dots. ACS Energy Lett. 2021, 6 (8), 2697–2703. 10.1021/acsenergylett.1c01067.

[ref27] Song W. S.; Lee H. S.; Lee J. C.; Jang D. S.; Choi Y.; Choi M.; Yang H. Amine-Derived Synthetic Approach to Color-Tunable InP/ZnS Quantum Dots with High Fluorescent Qualities. J. Nanopart. Res. 2013, 15, 1750 10.1007/s11051-013-1750-y.

[ref28] Tessier M. D.; Dupont D.; De Nolf K.; De Roo J.; Hens Z. Economic and Size-Tunable Synthesis of InP/ZnE (E = S, Se) Colloidal Quantum Dots. Chem. Mater. 2015, 27 (13), 4893–4898. 10.1021/acs.chemmater.5b02138.

[ref29] Panzer R.; Guhrenz C.; Haubold D.; Hubner R.; Gaponik N.; Eychmuller A.; Weigand J. J. Versatile Tri(pyrazolyl)phosphanes as Phosphorus Precursors for the Synthesis of Highly Emitting InP/ZnS Quantum Dots. Angew. Chem., Int. Ed. 2017, 56 (46), 14737–14742. 10.1002/anie.201705650. 28834116

[ref30] Shim H. S.; Ko M.; Nam S.; Oh J. H.; Jeong S.; Yang Y.; Park S. M.; Do Y. R.; Song J. K. InP/ZnSeS/ZnS Quantum Dots with High Quantum Yield and Color Purity for Display Devices. ACS Appl. Nano Mater. 2023, 6 (2), 1285–1294. 10.1021/acsanm.2c04936.

[ref31] Van Avermaet H.; Schiettecatte P.; Hinz S.; Giordano L.; Ferrari F.; Nayral C.; Delpech F.; Maultzsch J.; Lange H.; Hens Z. Full-Spectrum InP-Based Quantum Dots with Near-Unity Photoluminescence Quantum Efficiency. ACS Nano 2022, 16 (6), 9701–9712. 10.1021/acsnano.2c03138. 35709384

[ref32] Jo J. H.; Jo D. Y.; Lee S. H.; Yoon S. Y.; Lim H. B.; Lee B. J.; Do Y. R.; Yang H. InP-Based Quantum Dots Having an InP Core, Composition-Gradient ZnSeS Inner Shell, and ZnS Outer Shell with Sharp, Bright Emissivity, and Blue Absorptivity for Display Devices. ACS Appl. Nano Mater. 2020, 3 (2), 1972–1980. 10.1021/acsanm.0c00008.

[ref33] Buffard A.; Dreyfuss S.; Nadal B.; Heuclin H.; Xu X.; Patriarche G.; Mézailles N.; Dubertret B. Mechanistic Insight and Optimization of InP Nanocrystals Synthesized with Aminophosphines. Chem. Mater. 2016, 28 (16), 5925–5934. 10.1021/acs.chemmater.6b02456.

[ref34] Tessier M. D.; De Nolf K.; Dupont D.; Sinnaeve D.; De Roo J.; Hens Z. Aminophosphines: A Double Role in the Synthesis of Colloidal Indium Phosphide Quantum Dots. J. Am. Chem. Soc. 2016, 138 (18), 5923–5929. 10.1021/jacs.6b01254. 27111735

[ref35] Schrader E. Synthesis and Properties of Acylphosphines. Ph.D. Thesis, ETH Zurich: Switzerland, 2018.

[ref36] Wiesner T.; Neshchadin D.; Glotz G.; Gfader Z.; Schrader E.; Christen S.; Fischer R. C.; Kelterer A.; Gescheidt G.; Grützmacher H.; Haas M. Symmetrical and Mixed Tris(acyl)phosphines: Synthesis, Oxidation and Photochemistry. Chem. - Eur. J. 2023, 29 (67), e202302535 10.1002/chem.202302535. 37701996

[ref37] Scott D. J.; Cammarata J.; Schimpf M.; Wolf R. Synthesis of Monophosphines Directly from White Phosphorus. Nat. Chem. 2021, 13, 458–464. 10.1038/s41557-021-00657-7. 33820964

[ref38] Grützmacher H.; Pringle P. G.; Bispinghoff M. A Versatile Process for the Preparation of Acylphosphines. US 2019/0382424 A1, 2019.

[ref39] Green W. A. Industrial Photoinitiators: A Technical Guide. 1st ed.; CRC Press: 2010.

[ref40] Kowalska A.; Sokolowski J.; Bociong K. The Photoinitiators Used in Resin Based Dental Composite - A Review and Future Perspectives. Polymers (Basel, Switz.) 2021, 13 (3), 470 10.3390/polym13030470. PMC7867280 33540697

[ref41] Xie L.; Shen Y.; Franke D.; Sebastian V.; Bawendi M. G.; Jensen K. F. Characterization of Indium Phosphide Quantum Dot Growth Intermediates Using MALDI-TOF Mass Spectrometry. J. Am. Chem. Soc. 2016, 138 (41), 13469–13472. 10.1021/jacs.6b06468. 27690411

[ref42] Bispinghoff M. From Elemental Phosphorus to Functionalized Organophosphorus Compounds. Ph.D. Thesis, ETH Zurich: Switzerland, 2017.

[ref43] Gary D. C.; Cossairt B. M. Role of Acid in Precursor Conversion During InP Quantum Dot Synthesis. Chem. Mater. 2013, 25 (12), 2463–2469. 10.1021/cm401289j.

[ref44] Becker G.; Beck H. P. Bildung und Eigenschaften von Acylphosphinen. IV. Molekül- und KristallStruktur des Aluminium-tris(dibenzoylphosphid)s. Z. Anorg. Allg. Chem. 1977, 430 (1), 91–109. 10.1002/zaac.19774300109.

[ref45] Carpenter S. H.; Wolford N. J.; Billow B. S.; Fetrow T. V.; Cajiao N.; Radovic A.; Janicke M. T.; Neidig M. L.; Tondreau A. M. Homoleptic Uranium-Bis(acyl)phosphide Complexes. Inorg. Chem. 2022, 61 (32), 12508–12517. 10.1021/acs.inorgchem.2c00639. 35905438

[ref46] Chen J.; Carpenter S. H.; Fetrow T. V.; Mengell J.; Kirk M. L.; Tondreau A. M. Magnetism Studies of Bis(acyl)phosphide-Supported Eu^3+^ and Eu^2+^ Complexes. Inorg. Chem. 2022, 61 (46), 18466–18475. 10.1021/acs.inorgchem.2c02675. 36331515

[ref47] Carpenter S. H.; Mengell J.; Chen J.; Jones M. R.; Kirk M. L.; Tondreau A. M. Determining the Effects of Zero-Field Splitting and Magnetic Exchange in Dimeric Europium(II) Complexes. Inorg. Chem. 2024, 63 (19), 8516–8520. 10.1021/acs.inorgchem.4c00694. 38667056

[ref48] Madadi M.; Khalili Najafabadi B.; Fard M. A.; Corrigan J. F. NHC-Stabilized Bis(trimethylsilyl)phosphido Complexes of Pd^II^ and Ni^II^. Eur. J. Inorg. Chem. 2015, 2015 (19), 3094–3101. 10.1002/ejic.201500491.

[ref49] Weber L.; Reizig K.; Frebel M. Übergangsmetall-substituierte Acylphosphane und Phosphaalkene, IX. Zur Synthese von Phosphaalkenyl-, Mono- und Diacyl- phosphidokomplexen aus [Bis(trimethylsilyl)phosphido]-dicarbonyl(pentamethylcyclopentadienyl)eisen und Carbonsäurechloriden. Chem. Ber. 1986, 119 (6), 1857–1867. 10.1002/cber.19861190608.

[ref50] Weber L.; Bungardt D. Übergangsmetall-Substituierte Acylphosphane und Phosphaalkene: XI. Phosphaalkenyl-, Disilylphosphido- und Diacylphosphidokomplexe des Dicarbonylpentamethylcyclopentadienylosmium. Zur Kenntnis von [(η^5^-C_5_Me_5_)Os(CO)_2_]_2_. J. Organomet. Chem. 1986, 311 (3), 269–280. 10.1016/0022-328X(86)80249-2.

[ref51] Weber L.; Reizig K.; Boese R. Transition Metal Substituted Acylphosphines and Phosphaalkenes. 5. Synthesis and Structure of (C_5_Me_5_)(CO)_2_RuP[C(O)(*t*-Bu)]_2_, the First Diacylphosphido Complex with Metal-Phosphorus Coordination. Organometallics 1985, 4 (10), 1890–1891. 10.1021/om00129a034.

[ref52] Briand G. G.; Davidson R. J.; Decken A. Substituent Effects on Indium-Phosphorus Bonding in (4-RC_6_H_4_S)_3_In·PR’_3_ Adducts (R = H, Me, F; R’ = Et, Cy, Ph): A Spectroscopic, Structural, and Thermal Decomposition Study. Inorg. Chem. 2005, 44 (26), 9914–9920. 10.1021/ic051138r. 16363862

[ref53] Kirkwood N.; De Backer A.; Altantzis T.; Winckelmans N.; Longo A.; Antolinez F. V.; Rabouw F. T.; De Trizio L.; Geuchies J. J.; Mulder J. T.; Renaud N.; Bals S.; Manna L.; Houtepen A. J. Locating and Controlling the Zn Content in In(Zn)P Quantum Dots. Chem. Mater. 2020, 32 (1), 557–565. 10.1021/acs.chemmater.9b04407.

[ref54] Ubbink R. F.; Almeida G.; Iziyi H.; du Fosse I.; Verkleij R.; Ganapathy S.; van Eck E. R. H.; Houtepen A. J. A Water-Free In Situ HF Treatment for Ultrabright InP Quantum Dots. Chem. Mater. 2022, 34 (22), 10093–10103. 10.1021/acs.chemmater.2c02800. 36439318 PMC9686131

[ref55] Stam M.; Almeida G.; Ubbink R. F.; van der Poll L. M.; Vogel Y. B.; Chen H.; Giordano L.; Schiettecatte P.; Hens Z.; Houtepen A. J. Near-Unity Photoluminescence Quantum Yield of Core-Only InP Quantum Dots via a Simple Postsynthetic InF_3_ Treatment. ACS Nano 2024, 18 (22), 14685–14695. 10.1021/acsnano.4c03290. 38773944 PMC11155241

