# Supplementary material for: Acylphosphine Route to Colloidal InP Quantum Dots
Source: J Am Chem Soc. 2025 Mar 24;147(13):11446–55. doi: 10.1021/jacs.5c01305 (PMC11969539; doi:10.1021/jacs.5c01305)
Supplement: Supplementary file 1 — ja5c01305_si_001.pdf [file ja5c01305_si_001.pdf]

Supporting Information for

Acylphosphine Route to Colloidal InP Quantum Dots

Andriy Stelmakh,†‡ Georgios Marnieros,†‡ Erik Schrader,† Georgian Nedelcu,†‡ Oleh Hordiichuk,†‡ Eduard Rusanov,†‡ Ihor Cherniukh,†‡ Daniel Zindel,§ Hansjörg Grützmacher,† Maksym V. Kovalenko†‡#*

†Laboratory of Inorganic Chemistry, Department of Chemistry and Applied Biosciences, ETH Zürich, Vladimir Prelog Weg 1, CH-8093 Zürich, Switzerland

‡Laboratory for Thin Films and Photovoltaics, Empa – Swiss Federal Laboratories for Materials Science and Technology, Überlandstrasse 129, CH-8600 Dübendorf, Switzerland

§Laboratory of Physical Chemistry, Department of Chemistry and Applied Biosciences, ETH Zürich, Vladimir Prelog Weg 2, CH-8093 Zürich, Switzerland

#SKKU Institute of Energy Science and Technology (SIEST), Sungkyunkwan University (SKKU), 2066, Seobu-ro, Jangan-gu, Suwon, Gyeonggi-do 16419, Republic of Korea

*mvkovalenko@ethz.ch

Table of contents

Experimental details S2
- Chemicals S2
- General synthesis of acylphosphines S2
- Syntheses of $InNu_3$ compounds S4
- Syntheses of heteroleptic complexes **6-8** S5
- Additional details of the colloidal InP NCs syntheses S6
- X-ray crystallography S6
- Characterization techniques S7

Additional figures S9

Crystallographic data S30

Supporting references S33

Experimental details

Chemicals

Sodium metal (Na, 99.8%, Chemie Brunschwig AG), red phosphorus (P, ≥97.0%, Aldrich), naphthalene (for synthesis, Merck), indium powder (In, ~325 mesh, 99.99%, abcr), indium (III) chloride ($InCl_3$, anhydrous, 99.99% metals basis, abcr), indium acetate ($In(OAc)_3$, 99.99% metals basis, Aldrich), zinc acetate ($Zn(OAc)_2$, 99.99% metals basis, Aldrich), lithium bis(trimethylsilyl)amide ($LiN(SiMe_3)_2$, 97%, Aldrich), methyl benzoate (99%, Aldrich), methyl *p*-toluate (99%, Thermo Scientific), methyl 2-methylbenzoate (98%, Apollo Scientific), methyl trimethylacetate (99%, Aldrich), benzoyl chloride (≥99%, Aldrich), *p*-toluoyl chloride (98%, Fluorochem), *o*-toluoyl chloride (99%, Acros), trimethylacetyl chloride (99%, Aldrich), bis(2,4,6-trimethylbenzoyl)phosphine (^{Mes}BAPH, Nanjing Weifu Chemical Technology), benzoyl peroxide (97% dry wt., wet with 25% water, abcr), 4-methylpyridine (4-MePy, 99%, Acros), 2-methyl-2-propanethiol (^{t}BuSH, 99%, Aldrich), thiophenol (99+%, Alfa Aesar), 4-fluorothiophenol (98%, Apollo Scientific), *p*-tolyl disulfide (98%, Aldrich), bis(4-methoxyphenyl)disulfide (Apollo Scientific), triethylamine (Et_3N, anhydrous, 99%, Fluorochem), hydrogen peroxide (H_2O_2, >30% w/v, Fisher), formic acid (98.0-100%, Aldrich), citric acid (anhydrous, 99.5%, Acros), oleic acid (OLA, technical grade, 90%, Aldrich), myristic acid (HMy, Sigma grade, ≥99%, Sigma), oleylamine (OlAm, approx. C18-content 80-90%, nitrogen flushed, Acros), tri-*n*-octylphosphine (TOP, min. 97%, Strem), 1-octadecene (ODE, technical grade, 90%, Aldrich), Dowtherm^{TM} A (Diphyl, eutectic mixture of 26.5% diphenyl and 73.5% diphenyl oxide, Aldrich), tetrahydrofuran (THF, >99.9%, HPLC grade, Merck/Aldrich), *n*-hexane (Hex, >97%, HPLC grade, Aldrich), *n*-pentane (extra dry, 99+%, Thermo Scientific), toluene (≥99.7%, Aldrich), diethyl ether (Et_2O, 99.5%, Merck/Aldrich), acetone (≥99.5%, Aldrich), anhydrous acetone (extra dry, 99.8%, Thermo Scientific), ethanol (EtOH, absolute, >99.8%, Aldrich), methanol (>99.8%, VWR), dry methanol (max. 0.005% water, PanReac AppliChem), anhydrous acetonitrile (extra dry, over molecular sieve, 99.9%, Thermo Scientific), dimethoxyethane (DME, >99%, Aldrich), anhydrous *tert*-butanol (^{t}BuOH, 99.5%, Alfa Aesar), benzene-d6 (C_6D_6, 99.50 % D, Eurisotop), toluene-d8 (99.50 % D, Eurisotop), tetrahydrofuran-d8 (THF-d8, 99.50 % D, Eurisotop), Celite® Hyflo Super Cel® (Roth). Benzoyl peroxide was dried under vacuum for 2 h before use. 4-MePy was degassed by three freeze-pump-thaw cycles and then stored over 4Å molecular sieves. Dry ODE was obtained by bubbling the solvent with Ar gas at 120 °C for 2 h. Diphyl was dried under vacuum for 2 h at 100 °C. THF and Et_2O were dried using a SP-1 solvent purification system from LC Technology Solutions Inc. Et_2O was distilled prior to drying in order to remove the stabilizers. Toluene, *n*-hexane and ethanol were dried using a solvent purification system from Vigor Tech. DME was dried using an Innovative Technology PureSolv MD 7 solvent purification system. Deuterated solvents were dried with 4Å molecular sieves for at least 24 h. Deionized water was degassed by bubbling it with Ar gas for 2 h. Dry solvents and air-sensitive chemicals were stored inside of a nitrogen-filled glovebox. All other chemicals were used as received.

General synthesis of acylphosphines

Different BAPs and TAPs were synthesized according to a previously reported general procedure[1-3] with some modifications. Sodium metal (5 g, 217 mmol) was added in small chunks to a 500 mL two-neck flask containing dry DME (160 mL) and naphthalene (0.42 g, 3.3 mmol). The reaction mixture was stirred until it had turned deep green. Red phosphorus (2.08 g, 66.8 mmol) was added and the resulting suspension was stirred for 2 days at room temperature. Leftover sodium was removed with tweezers and the black suspension was cooled to 0 °C by means of an ice bath. Dry degassed *tert*-butanol (4.96 g, 66.8

mmol) was mixed with dry DME (20 mL) in a dropping funnel and subsequently added dropwise to the stirred reaction mixture. The ice bath was removed and the suspension was stirred for 2 h at room temperature. The suspension was again cooled to 0 °C and a methyl ester of a desired carboxylic acid (66.8 mmol) was added dropwise. The reaction mixture was stirred for 1 h at room temperature, while turning dark orange. The suspension was again cooled to 0°C and the corresponding acyl chloride (*ca.* 80-100 mmol for the synthesis of BAPs and *ca.* 180-200 mmol for the synthesis of TAPs) was added dropwise. The reaction mixture was stirred overnight at room temperature and the reaction was monitored with ^{31}P-NMR, evidencing formation of a sodium salt of BAP or TAP, respectively. In the case of BAPs, it is important to add slightly insufficient amounts of acyl chlorides in order to avoid formation of TAPs, which could be difficult to separate from the main products.

^{Ph}BAPH. Concentrated formic acid (7.6 mL, 200 mmol) was added dropwise and the resulting thick suspension was stirred overnight at room temperature. Solids were removed by filtration through celite and washed twice with 50 mL of THF. The combined filtrates were evaporated under vacuum and the residual was dissolved in a minimum amount of toluene (60-80 mL). The solution was filtered through a PTFE filter, concentrated under vacuum until the onset of crystallization and placed into a freezer (-15 °C) for several hours. The bright orange crystalline product was collected by decanting out the mother liquor, washing it three times with 10 mL of cold n-hexane, and drying it under a vacuum for 12 h. The spectral data matched those reported in literature.[3]

$^{p\text{-Tol}}$BAPH. Concentrated formic acid (7.6 mL, 200 mmol) was added dropwise and the resulting thick suspension was stirred overnight at room temperature. Solids were removed by filtration through celite and washed three times with 100 mL of THF. The combined filtrates were concentrated under vacuum until the onset of crystallization and the bright orange crystalline product was further precipitated by adding two-fold excess of EtOH. The product was collected by filtration through a Schlenk frit, washed twice with 20 mL of EtOH and dried under vacuum for 12 h. ^{1}H-NMR (C_6D_6, 300 MHz): δ = 20.30 (d, $^3J_{PH}$ = 3.3 Hz, 1H, OH), 8.16 (d, 4H, *o*-H_{enol}), 6.83 (d, 4H, *m*-H_{enol}), 1.94 (s, 6H, $CH_{3,enol}$) ppm. ^{31}P-NMR (C_6D_6, 121.5 MHz): δ = 56.9 (s, enol) ppm.

$^{o\text{-Tol}}$BAPH. Concentrated formic acid (7.6 mL, 200 mmol) was added dropwise and the resulting thick suspension was stirred overnight at room temperature. Solids were removed by filtration through celite and washed twice with 50 mL of THF. The filtrates were combined and the orange product was precipitated by a slow addition of degassed H_2O. The product was collected by filtration through a Schlenk frit, washed twice with 20 mL of EtOH/H_2O (1:1 v/v) mixture and dried under vacuum for 2 days. ^{1}H-NMR (C_6D_6, 300 MHz): δ = 19.77 (d, $^3J_{PH}$ = 2.4 Hz, 1H, OH), 7.95 (d, 2H, *o*-H_{enol}), 7.63 (d, 2H, *o*-H_{keto}), 7.03-6.78 (m, 6H, *m*-H and *p*-H), 5.50 (d, $^1J_{PH}$ = 245.7 Hz, 1H, PH), 2.47 (s, 6H, $CH_{3,enol}$), 2.35 (s, 6H, $CH_{3,keto}$) ppm. ^{31}P-NMR (C_6D_6, 121.5 MHz): δ = 73.6 (s, enol), 11.8 (d, $^1J_{PH}$ = 245.4 Hz, keto) ppm.

$^{t\text{-Bu}}$BAPH. Concentrated formic acid (7.6 mL, 200 mmol) was added dropwise and the resulting thick suspension was stirred overnight at room temperature. Solids were removed by filtration through celite and washed twice with 50 mL of THF. The filtrates were combined and evaporated under vacuum. The residual was dissolved in a minimum amount of *n*-pentane, filtered through a PTFE filter and stored in a freezer (-35 °C). Nearly colorless crystals of the product were collected by decanting out the mother liquor, washed with cold *n*-pentane and dried under vacuum for 1 h. The spectral data matched those reported in literature.[4]

^{Ph}TAP. THF (80 mL) was added to the greenish-yellow reaction mixture and the solids were removed by filtration through celite. The filtrate was concentrated under vacuum to *ca.* 50 mL (yellow needles start to crystallize) and the product was further precipitated by adding 100 mL of *n*-hexane. The product was collected by filtration through a Schlenk frit, washed with 40 mL of *n*-hexane and dried under vacuum for 8 h. The spectral data matched those reported in literature.[5]

Typical yields of the isolated acylphosphines – 40-90%.

Syntheses of $InNu_3$ compounds

$In[N(TMS)_2]_3$. Indium tris[bis(trimethylsilyl)amide] was prepared according to Bürger et al.[6] $InCl_3$ (11.77 g, 53.3 mmol) was dissolved in refluxing Et_2O (650 mL), followed by the dropwise addition of $LiN(SiMe_3)_2$ (26.77 g, 160 mmol) dissolved in 300 mL of Et_2O. The reaction mixture was left refluxing for 24 h and the solids were separated by filtration through celite. The filtrate evaporated under vacuum until the onset of crystallization and the suspension was cooled to -10 °C to trigger further precipitation. The colorless crystalline product was collected by filtration through a Schlenk frit, recrystallized from Et_2O at -10 °C, and dried under vacuum for 4 h. Yield – 20.1 g (63 %). The spectral and X-ray diffraction data matched those reported in the literature.[7-8] ^{1}H-NMR (C_6D_6, 300 MHz): δ = 0.34 (s, 54H) ppm.

$[In(OOCPh)_3(4\text{-}MePy)_2]\cdot(4\text{-}MePy)$. Monomeric indium benzoate complex was synthesized according to Andras et al.[9] A mixture of 325-mesh indium powder (1.0 g, 8.7 mmol) and benzoyl peroxide (3.16 g, 13.05 mmol) in 70 mL of 4-methylpyridine was stirred under nitrogen at room temperature for 6 days. *n*-hexane (400 mL) was added and the mixture was filtered through a Schlenk frit. The resulting off-white solid was washed three times with 50 mL of *n*-hexane. The product was recrystallized by dissolving it in hot 4-methylpyridine (80 mL, 80 °C), filtering the obtained hot solution and precipitating the product by *n*-hexane (200 mL). The colorless crystalline product was collected by filtration and dried under vacuum for 12 h. An adduct with one co-crystallized 4-MePy molecule was obtained instead of the reported hydrated structure. Yield – 5.61 g (85%). ^{1}H-NMR (C_6D_6, 300 MHz): δ = 8.77 (d, 6H, $Ar_{4\text{-}MePy}$), 8.48 (m, 6H, *o*-Ph), 7.07 (m, 9H, *m*-Ph and *p*-Ph), 6.45 (d, 6H, $Ar_{4\text{-}MePy}$), 1.63 (s, 9H, CH_3) ppm.

$[In(O^tBu)_3]_2$. Indium *tert*-butoxide was synthesized according to Veith et al.[10] The spectral data matched those reported in literature.

$[In(S^tBu)_3]_2$. Indium *tert*-butylthiolate was synthesized according to Suh et al.[11] The spectral data matched those reported in literature.

Indium arylthiolates

Thiophenol and *para*-fluorothiophenol were converted into the corresponding disulfides by oxidation with aqueous H_2O_2.[12] Indium arylthiolates were prepared from the corresponding aryl disulfides according to Briand et al.[12] with slight modifications. A solution of aryl disulfide (15 mmol) in warm methanol (60 mL) was added dropwise to a suspension of indium powder (1.15 g, 10 mmol) in methanol (25 mL). The reaction mixture was refluxed for 3 h and then cooled down to room temperature.

$In(SPh)_3$. Solids were removed by filtration through a PTFE filter and the obtained filtrate was evaporated under vacuum, resulting in a white powder. The product was washed three times with 20 mL of *n*-hexane and dried under vacuum for 5 h. Yield – 3.95 g (89 %). ^{1}H-NMR (THF-d8, 300 MHz): δ = 7.43 (d, 6H, *o*-Ph), 7.10 (t, 6H, *m*-Ph), 7.02 (t, 3H, *p*-Ph) ppm.

In(SPh-F-*p*)$_3$. Solids were removed by filtration and washed with 40 mL of hot methanol. The combined filtrates were evaporated under vacuum, resulting in a white powder. The product was washed three times with 20 mL of *n*-hexane and dried under vacuum for 3 h. Yield – 3.5 g (70 %). ^{1}H-NMR (THF-d8, 300 MHz): δ = 7.39 (dd, $^4J_{FH}$ = 5.1 Hz, $^3J_{HH}$ = 8.7 Hz, 6H, *o*-Ph), 7.10 (dd, $^3J_{FH}$ = 8.7 Hz, $^3J_{HH}$ = 8.7 Hz, 6H, *m*-Ph) ppm.

In(SPh-Me-*p*)$_3$. Solids were collected by filtration and dissolved in THF. The obtained solution was filtered and then evaporated under vacuum, resulting in a white powder. The product was washed three times with 20 mL of *n*-hexane and dried under vacuum for 3 h. Yield – 4.0 g (83 %). ^{1}H-NMR (THF-d8, 300 MHz): δ = 7.29 (d, 6H, Ar), 6.91 (d, 6H, Ar), 2.23 (s, 9H, CH_3) ppm.

In(SPh-OMe-*p*)$_3$. Solids were collected by filtration and dissolved in THF. The obtained solution was filtered and then evaporated under vacuum, resulting in a white powder. The product was washed three times with 20 mL of *n*-hexane and dried under vacuum for 3 h. Yield – 2.78 g (52 %). ^{1}H-NMR (THF-d8, 300 MHz): δ = 7.29 (d, 6H, Ar), 6.68 (d, 6H, Ar), 3.70 (s, 9H, CH_3) ppm.

Syntheses of heteroleptic complexes 6-8

[In$_2$(O^{t}Bu)$_4$(^{Mes}BAP)$_2$]·^{t}BuOH·½Hex (6·^{t}BuOH·½Hex). Solution of ^{Mes}BAPH (653 mg, 2 mmol) in THF (2 mL) was added dropwise to a solution of [In(O^{t}Bu)$_3$]$_2$ (668 mg, 1 mmol) in THF (20 mL) and the reaction mixture was stirred for 2 h at room temperature. The solvent was removed under vacuum, and the residual was dissolved in 5 mL of *n*-hexane. The resulting oversaturated solution was kept at room temperature for 1 day, leading to the formation of yellow crystals of **6**·^{t}BuOH·½Hex. The crystals were collected by filtration, washed twice with 1 mL of *n*-hexane and dried under vacuum for 2 h. Yield – 730 mg (60%). ^{1}H-NMR (C_6D_6, 300 MHz): δ = 6.67 (s, 8H, Ar), 2.58 (s, 24H, *o*-CH_3), 2.03 (s, 12H *p*-CH_3), 1.59 (s, 18H, O^{t}Bu), 1.51 (s, 18H, O^{t}Bu), 1.24 (m, 4H, *n*-hexane), 1.05 (s, 9H, ^{t}BuOH), 0.89 (t, 3H, *n*-hexane), 0.60 (s, 1H, ^{t}BuOH) ppm. ^{31}P{^{1}H}-NMR (C_6D_6, 121.5 MHz): δ = 100.4 (s) ppm.

[In$_2$(S^{t}Bu)$_4$(^{Ph}BAP)$_2$]·½THF·½Hex (7·½THF·½Hex). Solution of ^{Ph}TAP (69.2 mg, 0.2 mmol) in THF (1 mL) was added dropwise to a solution of [In(S^{t}Bu)$_3$]$_2$ (76.5 mg, 0.1 mmol) in THF (2 mL) and the reaction mixture was stirred for 12 h at room temperature. The resulting orange solution was filtered through a PTFE filter and concentrated (3x) under vacuum. Orange-red crystalline product was precipitated by adding *n*-hexane, collected by decanting out the mother liquor and dried under vacuum for 2 h. ^{1}H-NMR (C_6D_6, 300 MHz): δ = 8.39 (m, 8H, *o*-H), 7.10-6.87 (m, 12H, *m*-H, *p*-H), 3.58 (m, 2H, THF), 1.74 (br, 36H, S^{t}Bu), 1.42 (m, 2H, THF), 1.23 (m, 4H, *n*-hexane), 0.89 (t, 3H, *n*-hexane) ppm. ^{31}P{^{1}H}-NMR (C_6D_6, 121.5 MHz): δ = 72.4 (s) ppm.

[Et$_3$NH][InCl$_2$(^{Ph}BAP)$_2$] (8). Solution of ^{Ph}BAPH (30.3 mg, 0.125 mmol) in a mixture of THF and acetonitrile (1:1, 0.25 mL) was added to a solution of $InCl_3$ (27.7 mg, 0.125 mmol) in acetonitrile (1 mL), followed by the addition of triethylamine (26.1 μL, 0.188 mmol). The reaction mixture was stirred at 70 °C for 4 h. Solvent was evaporated under vacuum and the residual was extracted with toluene (2 mL). The extract was evaporated under vacuum to half of the original volume and stored at room temperature for several days, resulting in the formation of orange crystals that were suitable for X-ray diffraction experiments.

Additional details of the colloidal InP NCs syntheses

Preparation of In(My)$_3$ stock solution

Indium myristate stock solution was prepared following the same steps as described for the preparation of In(OLA)$_3$ in the main text, except HMy (4.385 g, 19.2 mmol) was used.

Hot-injection syntheses of InP QDs following Route 2

An empty 25 mL three-neck flask was equipped with a thermocouple and a rubber septum and connected to a Schlenk line through a reflux condenser. The atmosphere in the flask was exchanged by three cycles of consecutive switching from vacuum to argon gas. In(OLA)$_3$ (or Zn(OLA)$_2$) stock solution in ODE (1.8 mL, 0.2 M) was added into the flask and further diluted with dry degassed ODE to a total volume of 6 mL. The flask was then heated to 285 °C. In parallel, an injection solution was prepared by dissolving ^{Ph}TAP (83.0 mg, 0.24 mmol) and In(SPh)$_3$ (106.2 mg, 0.24 mmol) in Diphyl (1 mL) at 125 °C for 30 min, leading to the formation of wine-red In-^{Ph}MAP intermediate. The obtained solution was quickly injected into the reaction flask, and the thermocontroller was set at 275 °C. After 1 h, the reaction was quenched by rapid cooling to room temperature, and the contents were transferred into a glovebox to purify the NCs. The NCs precipitate from the crude reaction mixtures at room temperature. Yet, stable colloidal dispersions in *n*-hexane were obtained following the purification steps that are described in the main text.

Table S1. Additional details of the InP QDs syntheses presented in **Figure 4**.

R in In(^{R}BAP)$_3$	Ar in In(SAr)$_3$	Ligand	Reaction time	Isolated yield [a], %
Ph	Ph	Zn(OLA)$_2$	1 h	40
o-Tol	Ph	Zn(OLA)$_2$	2 h	50
Ph	Ph	In(OLA)$_3$	1 h	80
p-Tol	Ph	In(OLA)$_3$	20 min	50
o-Tol	*p*-MeO-Ph	In(OLA)$_3$	1 h	40
t-Bu	Ph	In(OLA)$_3$	1 h	60
Mes	*p*-F-Ph	In(OLA)$_3$	1 h	70

[a]Isolated yields were estimated by optically determining concentration of InP in the purified samples (sequentially diluted 41- and 9-fold by toluene) using molar extinction coefficient of InP at 310 nm.[13]

X-ray crystallography

Crystals of **1**, **2**, **3**, **5** and **7** were obtained from solutions in THF by precipitation with *n*-hexane at room temperature. Crystals of **6** were obtained from a solution in THF/*n*-hexane mixture at -5 °C. Crystals of **4** were obtained from a solution in Et$_2$O by cooling the solution to -35 °C. Crystals of In(SPh-F-*p*)$_3$ were obtained from a solution in methanol by slow evaporation.

The diffraction data for all single crystal samples were collected at 100K on a Rigaku XtaLAB Synergy-S diffractometer equipped with a PhotonJet-S microfocus Cu X-ray (λ = 1.54184 Å) source with a mirror design and hybrid photon counting HyPix-6000HE detector. The data were corrected for Lorentz-polarization effects. Absorption corrections from the equivalents with the multi-scan method were applied for all compounds. The processing procedures for the experiments to a resolution of 0.78Å were followed by procedures implemented in CrysAlisPro software.[14] The structures were solved by direct methods and refined by the full-matrix least-squares on F^2 for all reflections using SHELXS, SHELXT and SHELXL[15] operated under OLEX2.[16] The non-hydrogen atoms were refined with anisotropic displacement parameters. All CH atoms were placed at calculated positions and refined as riding, with default distances and Uiso(H). The OH and NH hydrogen atoms were found in DF synthesis of electron density and refined isotropically. Structures **4** (two independent and disordered molecules in asymmetric unit) and **5** (three independent molecules in asymmetric unit) were refined as nonmerohedral twins after treatment and data processing with hklf 5 using CrysAlisPro and OLEX2 software. Structure **4** was refined as a two-component twin with BASF coefficient 0.1783, while structure **5** was refined as a three-component twin with BASF coefficients for domains 0.3405 and 0.218. In structure **3** one of the *o*-MePh substituents in the ligand is disordered over two positions A and B with occupancies 0.86 and 0.14 respectively. In structure **4** we found two independent molecules in an asymmetric unit. In each of the independent molecules, one ligand is refined with full occupancy, while the two of them are disordered over two positions A and B with occupancies of 0.75 and 0.25 respectively in one molecule, and 0.63 and 0.37 respectively in the second. Since the occupancy of position B is relatively low, SADI and EADP restraints were applied to the bond distances and atomic displacement parameters during the refinement of **3** and **4**.

The crystal structure of compound **6** contains *n*-hexane molecule positioned at a centre of inversion, while the *t*-BuOH molecule occupies the general position. In contrast, compound **7** features alternating THF and *n*-hexane solvent molecules occupying channels along the crystallographic a-axis. Notably, each solvent molecule has a 50% occupancy, indicating equal probability of finding either THF or *n*-hexane at a specific position. To account for the disordered part in solvent molecules and refine its positions in compounds **6** and **7**, restraints and constraints were employed for the solvent atoms during the refinement process.

The crystal data for all compounds studied by X-ray single crystal diffraction experiments are provided in **Tables S2-S4**. Molecular structures of complexes **1-8** are visualized in **Scheme 1** and **Figure S4**. Polymeric structure of In(SPh-F-*p*)$_3$ is visualized in **Figure S16**.

Crystallographic data for all new structures that are mentioned in this paper have been deposited at Cambridge Crystallographic Data Centre as supplementary publication numbers CCDC 2406125-2406133. Copies of the data can be obtained, free of charge, on application to CCDC, 12 Union Road, Cambridge CB21EZ, UK, (fax: +44-(0)1223-336033 or e-mail: deposit@ccdc.cam.ac.uk).

Characterization techniques

Solution ^{1}H and ^{31}P NMR spectra were recorded at 7 T (300 and 121.5 MHz) using a Bruker AVANCE III HD spectrometer with a 5 mm PABBO probe. Chemical shifts were referenced using the ^{2}H signal of the solvent. Variable-temperature ^{31}P NMR experiments were performed at 11.7 T (202.5 MHz) using AVANCE III HD spectrometer with a 5 mm PABBO probe and a BCU II variable temperature unit. Temperatures were measured with a thermocouple near the sample, previously calibrated using ethylene glycol. Samples for optical characterization were diluted with toluene and placed into glass cuvettes with

1 cm path length. UV–visible light absorption spectra were recorded with a Jasco V-670 spectrophotometer using pure solvent as a reference. TEM samples were prepared by drop-casting the dilute solutions of the nanomaterials in *n*-hexane on ultrathin carbon-coated 400 mesh copper grids. STEM images were obtained using Jeol JEM-F200 and JEM-2200FS microscopes operating at 200 kV. Analytical investigations were done with two energy-dispersive X-ray spectrometers (EDXS, JEOL) attached to the JEM-F200 microscope column. X-ray diffraction (XRD) patterns of powders obtained in solvothermal experiments were acquired with Stoe IPDS II diffractometer (Cu Kα radiation) with an in-house modification enabling powder diffraction on samples with nanosized crystallites. Powder XRD data of colloidal InP NCs samples prepared by drop casting on mylar films were collected in transmission mode on a Stoe STADI P diffractometer equipped with a Dectris Mythen 1K detector and a focussing Ge-monochromator (CuKα1-radiation, λ =1.54060 Å). Rietveld refinement was performed with the help of GSAS-II software[17] using zinc-blende InP crystal structure (ICSD #41443) without structural modifications. The instrumental peak profile parameters were determined using a powder pattern of a silicon sample (NBS NIST 640c). Unit cell, isotropic In and P thermal parameters, crystallite size and proportion of Gaussian and Lorentzian contributions to the peak broadening (LGmix) were refined, while the background was approximated using Chebyshev polynomials with 9 coefficients. In the case of the smallest 2.1 nm NCs, an additional peak was included into the background to account for strong scattering from ligands around $2\theta = 20°$.[18] Atmospheric-pressure chemical ionization mass spectra (APCI-MS) were acquired with Advion ExpressIon CMS L instrument using direct inlet with a glass syringe via 5 μL loop. THF/acetonitrile (extra dry, 2:1 v/v) mixture without any additives was used as a solvent. The ionization was carried out using low fragmentation mode with a capillary temperature of 250 °C, a voltage of 180 V with an offset of 20 V and a source span of 30 V. The gas temperature was 350 °C (nitrogen). Positive and negative modes were acquired simultaneously with a mass range of 200-2000 m/z within 500 ms.

Additional figures

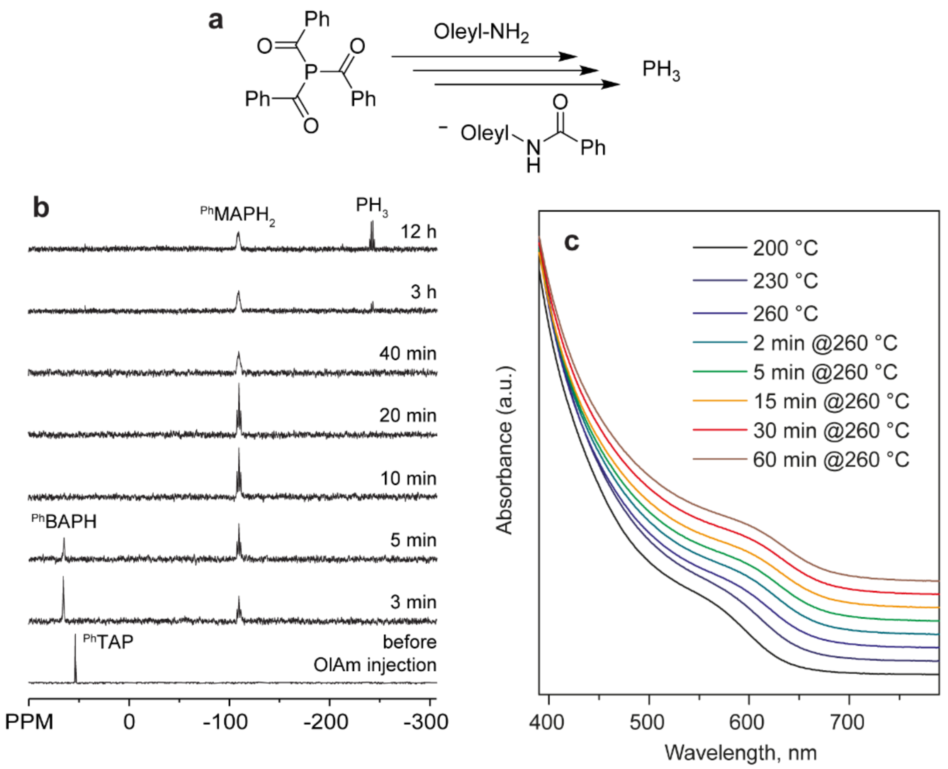

Figure S1. (**a**,**b**) Reaction of ^{Ph}TAP with OlAm leading to its rapid conversion into ^{Ph}BAPH, then into ^{Ph}MAPH$_2$ and eventually into PH_3 already at room temperature (**a**), as attested by the results of ^{31}P-NMR spectroscopy in C_6D_6 (**b**). ^{Ph}TAP and OlAm were combined in a 1:3 molar ratio. (**c**) Synthesis of InP NCs from ^{Ph}TAP and In(My)$_3$ in the presence of OlAm. In(My)$_3$ stock solution (2 mL, 0.2 M), dry degassed ODE (6 mL) and OlAm (0.6 mmol) were loaded into a 25 mL three-neck flask and heated to 120 °C under vacuum. The reaction mixture was degassed for 1 h and refilled with Ar gas. Then, a solution of ^{Ph}TAP (0.2 mmol) in Diphyl (2 mL) was injected, and the reaction mixture was rapidly heated to 260 °C. Formation of polydisperse InP NCs is evident from the appearance and gradual red shift of a broad shoulder around 550-600 nm in the absorption spectra of the reaction aliquots.

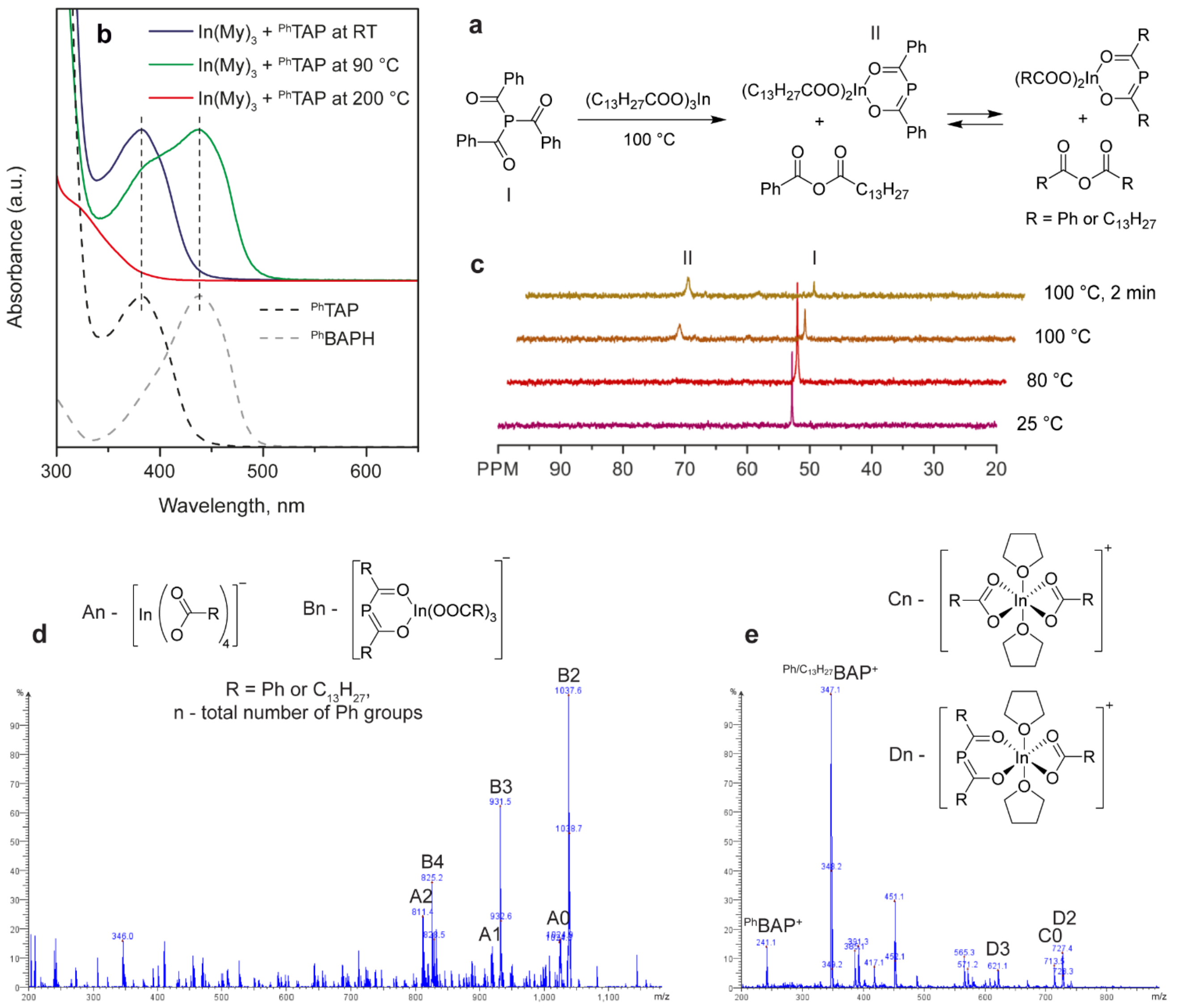

Figure S2. (**a-c**) Reaction of ^{Ph}TAP with In(My)$_3$ (in 1:4 molar ratio) in ODE that leads to a partial and reversible deacylation of ^{Ph}TAP with the formation of In-BAP complexes (**a**), as evidenced by the appearance of an absorption band around 440 nm in the optical absorption spectrum after heating to 90 °C (**b**) and a new signal at 74 ppm in the ^{31}P{^{1}H}-NMR spectra at 100 °C (**c**). Both features are characteristic of the ^{Ph}BAP anion. ^{Ph}BAP species disappear at higher temperatures, but the formation of InP is not observed. (**d**,**e**) Negative-ion (**d**) and positive-ion (**e**) atmospheric-pressure chemical ionization mass spectra (APCI-MS) of the reaction mixture with 1:2 molar ratio (diluted in 2:1 THF/acetonitrile). Different negatively and positively charged heteroleptic In-BAP complexes B and D are observed along with the corresponding pure carboxylates A and C. Due to a reversible nature of the reaction (see panel **a**), each complex family consists of several members that differ by the composition of their acyl groups (benzoyl or myristoyl).

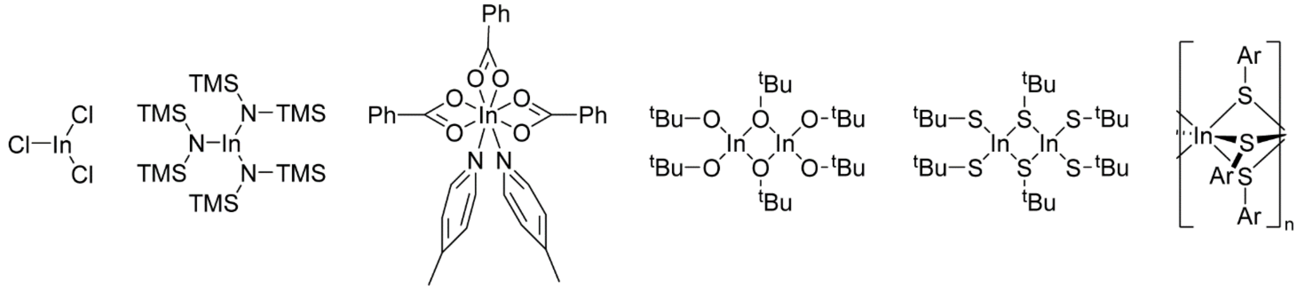

Figure S3. Molecular $InNu_3$ compounds that were tested in the reactions with complexes **1** and **5**.

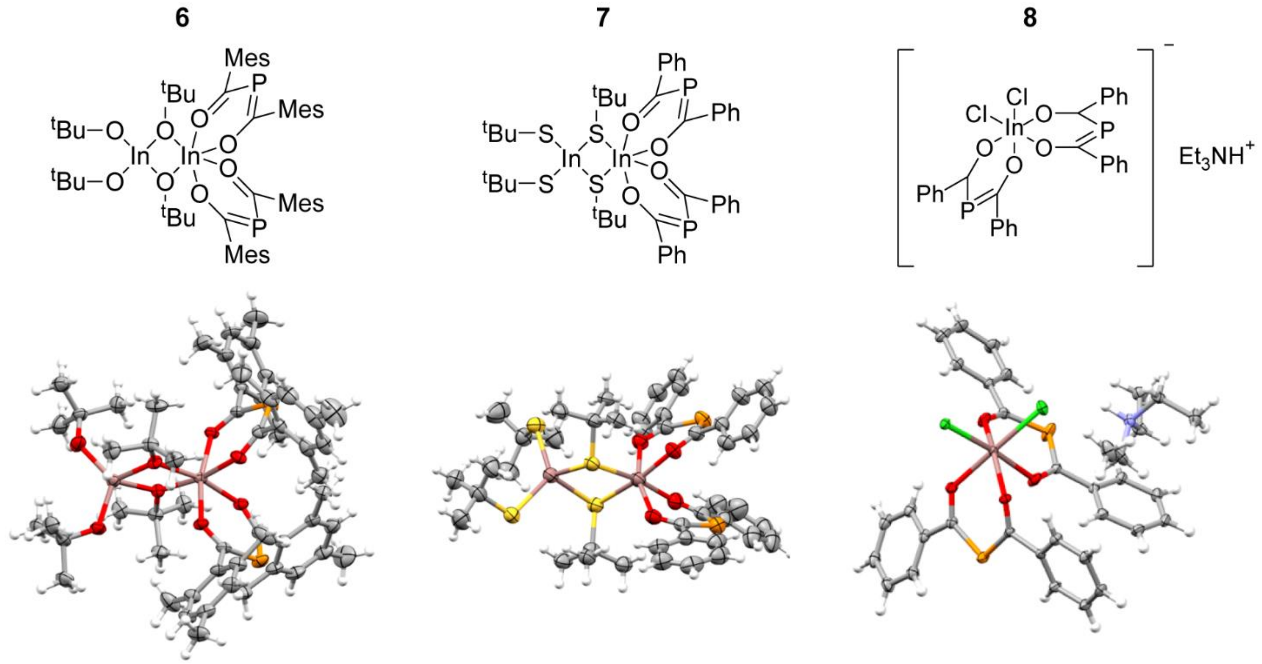

Figure S4. Molecular structures of some isolated heteroleptic In-Nu-BAP complexes in the solid state. Co-crystallized solvent molecules in **6** and **7** are omitted for clarity.

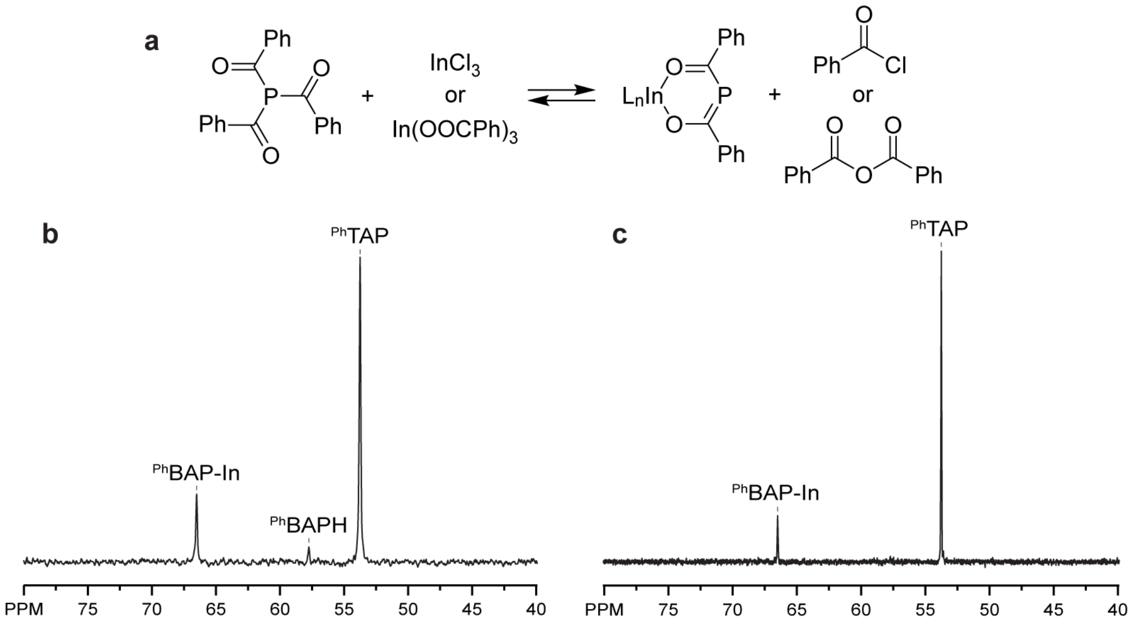

Figure S5. (**a**) Reaction scheme illustrating reversible deacylation of ^{Ph}TAP by $InCl_3$ or indium benzoate (L = ligand, which could be Cl^-, $PhCOO^-$, $^{Ph}BAP^-$ or THF). (**b**,**c**) ^{31}P-NMR spectra of the reaction mixtures with $InCl_3$ (**b**) and $[In(OOCPh)_3(4\text{-}MePy)_2]\cdot(4\text{-}MePy)$ (**c**) in THF after 1 h at 70 °C. ^{Ph}TAP (0.1 mmol) was dissolved in THF (0.5 mL) and added to a solution of $InCl_3$ or $[In(OOCPh)_3(4\text{-}MePy)_2]\cdot(4\text{-}MePy)$ (0.1 mmol) in THF (1 mL). The reaction mixture was then stirred inside a closed vial at 70 °C for 1 h leading to a color change from yellow to orange-red. ^{31}P-NMR spectra of the reaction aliquots indicate the formation of In-^{Ph}BAP complexes. In the case of indium benzoate, evaporation of the reaction mixture and dissolution of the residual in C_6D_6 led to a nearly complete reversal of the reaction towards the starting yellow ^{Ph}TAP.

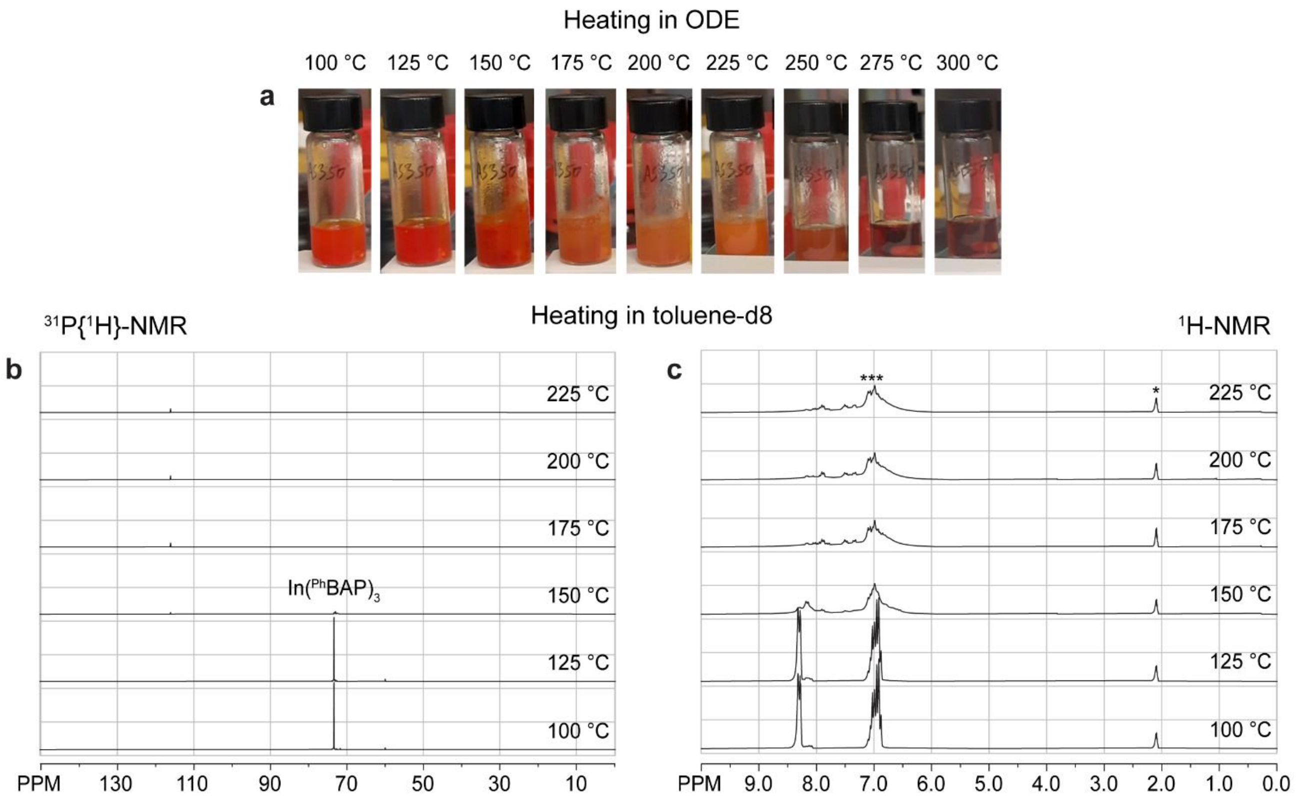

Figure S6. Solvothermal decomposition of complex **1** in ODE (**a**) and in toluene-d8 (**b**,**c**). In both cases, complex **1** decomposes at around 150 °C, as evidenced by the formation of orange precipitate (**a**) and by the disappearance of the In(^{Ph}BAP)$_3$ signals in ^{31}P{^{1}H}-NMR (**b**) and ^{1}H-NMR (**c**). *Residual solvent peaks.

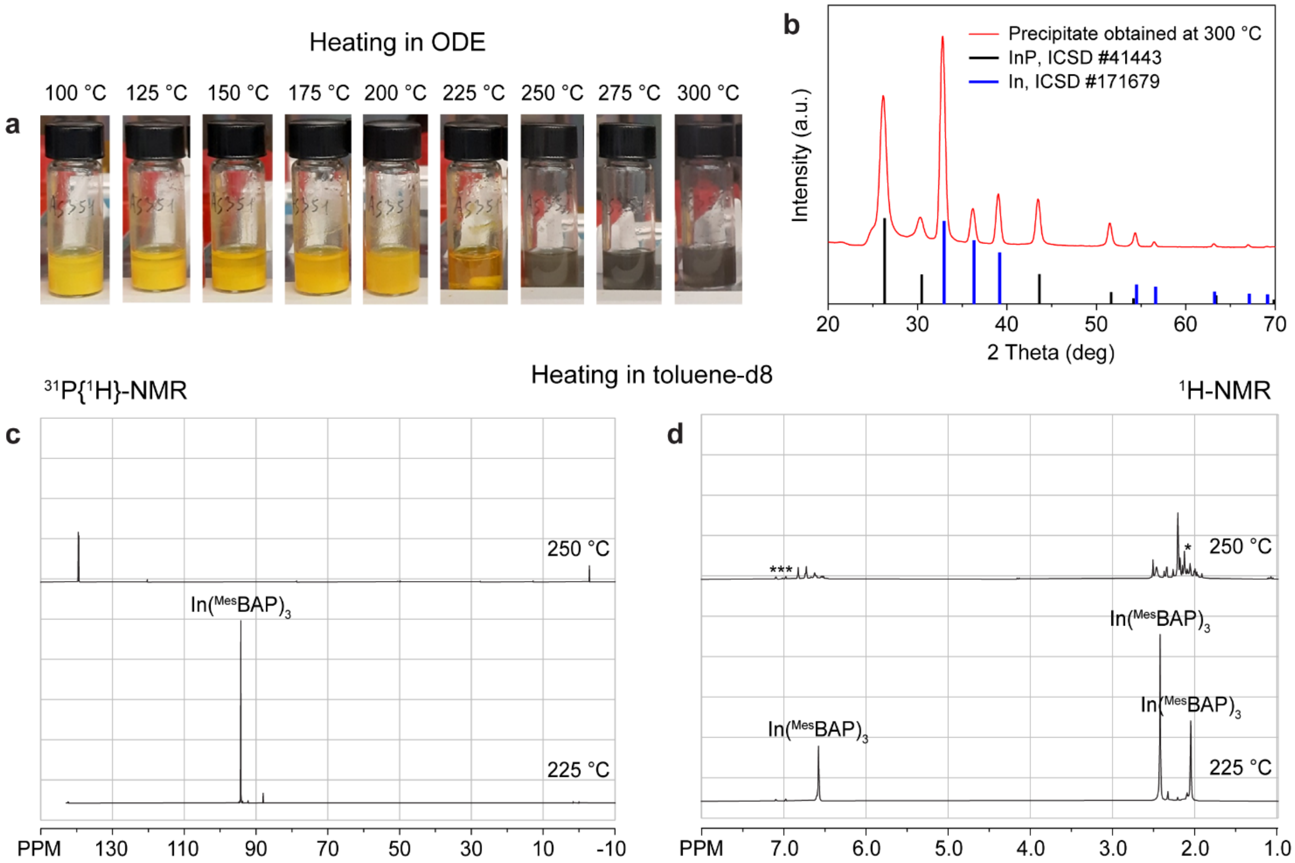

Figure S7. Solvothermal decomposition of complex **5** in ODE (**a**,**b**) and in toluene-d8 (**c**,**d**). In both cases, complex **5** decomposes at around 250 °C, as evidenced by the formation of a black precipitate (**a**) that contains metallic In and InP (**b**), and by the disappearance of the In(^{Mes}BAP)$_3$ signals in ^{31}P{^{1}H}-NMR (**c**) and ^{1}H-NMR (**d**). The appearance of a doublet around 139 ppm (indicative of a phosphorus-phosphorus bond) and the formation of metallic indium suggest homolysis as a main decomposition pathway. *Residual solvent peaks.

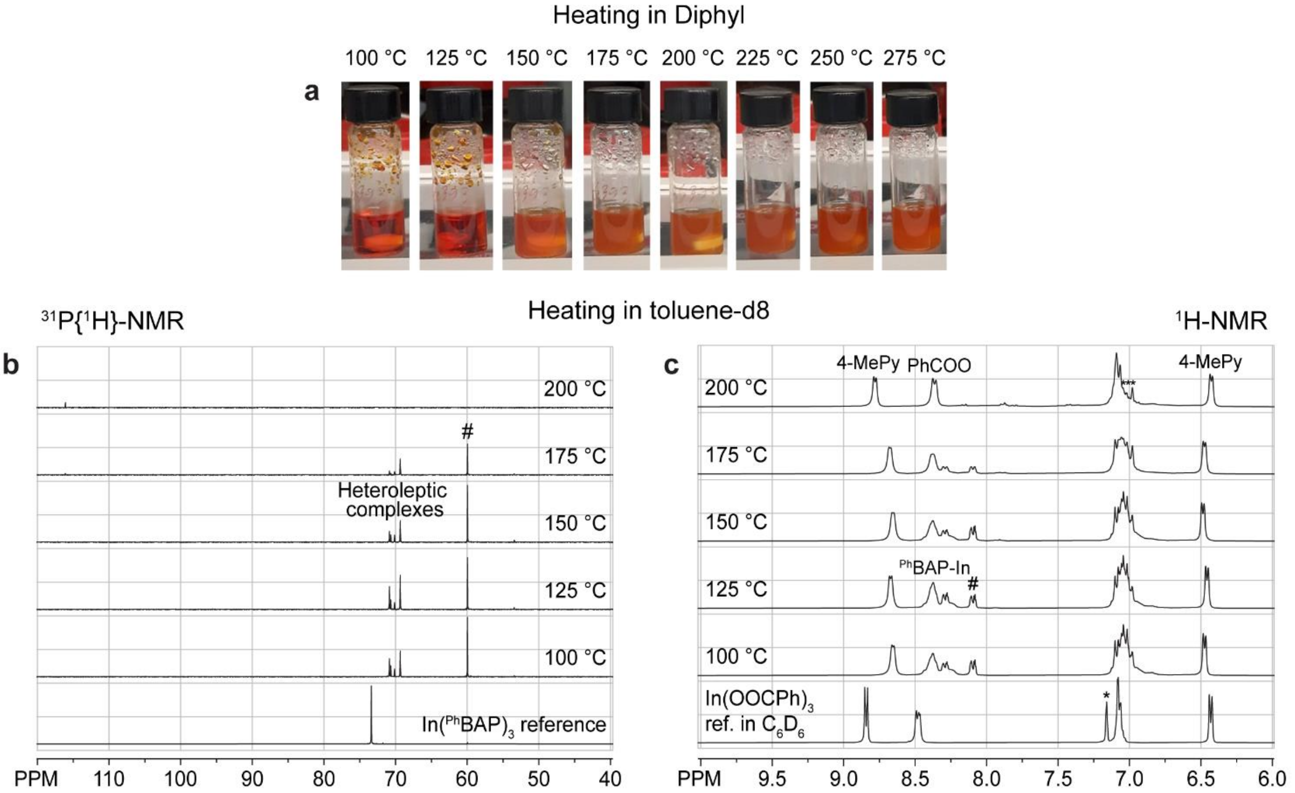

Figure S8. Heating of complex **1** with two equivalents of $[In(OOCPh)_3(4\text{-}MePy)_2]\cdot(4\text{-}MePy)$ in Diphyl (**a**) and in toluene-d8 (**b**,**c**). In-^{Ph}BAP complexes self-decompose at *ca.* 150 °C (**a**) and *ca.* 175 °C (**b**,**c**), leaving the starting indium benzoate intact (**c**). Hash sign (#) denotes ^{Ph}BAPH impurity that is caused by incomplete dryness of toluene-d8. Asterisk (*) denotes residual solvent peaks.

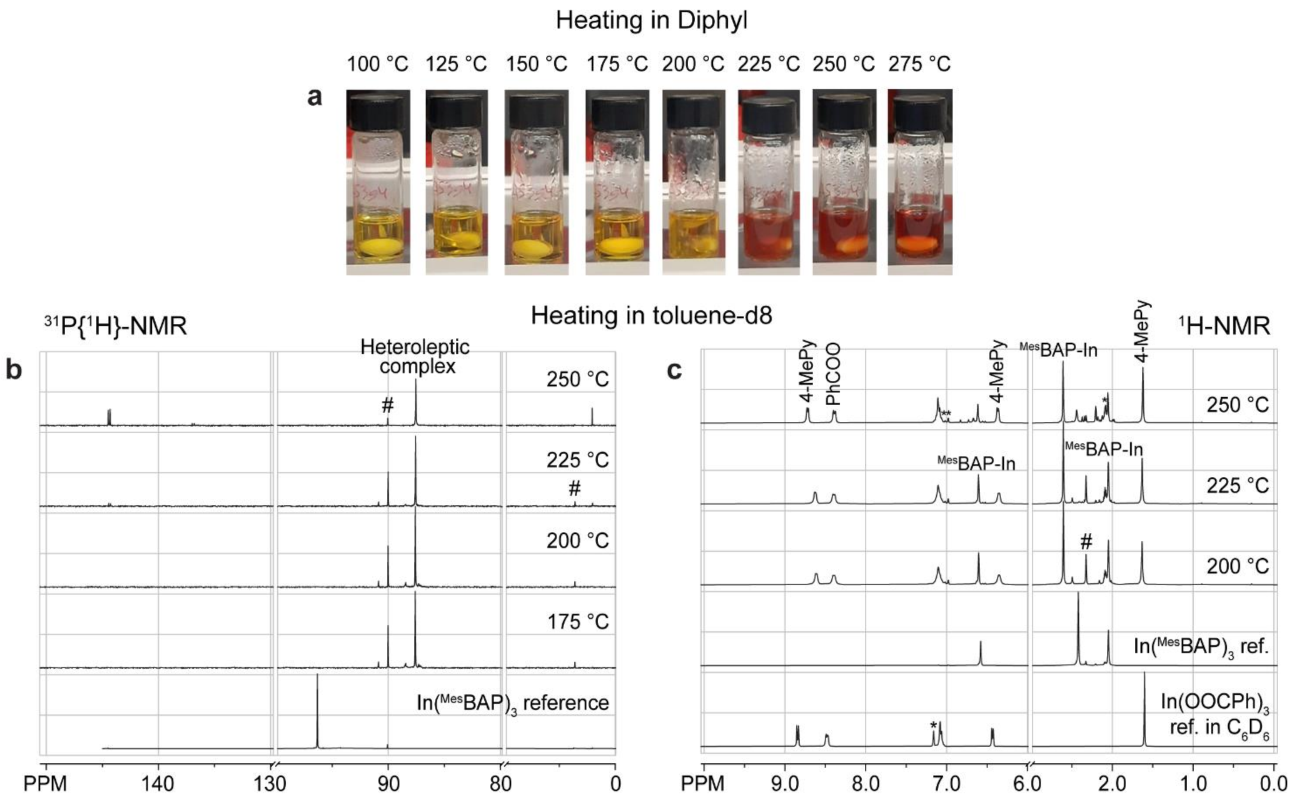

Figure S9. Heating of complex **5** with two equivalents of [In(OOCPh)$_3$(4-MePy)$_2$]·(4-MePy) in Diphyl (**a**) and in toluene-d8 (**b**,**c**). In-^{Mes}BAP complexes self-decompose at *ca.* 225 °C (**a**) and *ca.* 250 °C (**b**,**c**), leaving the starting indium benzoate intact (**c**). Hash sign (#) denotes ^{Mes}BAPH impurity that is caused by incomplete dryness of toluene-d8. Asterisk (*) denotes residual solvent peaks.

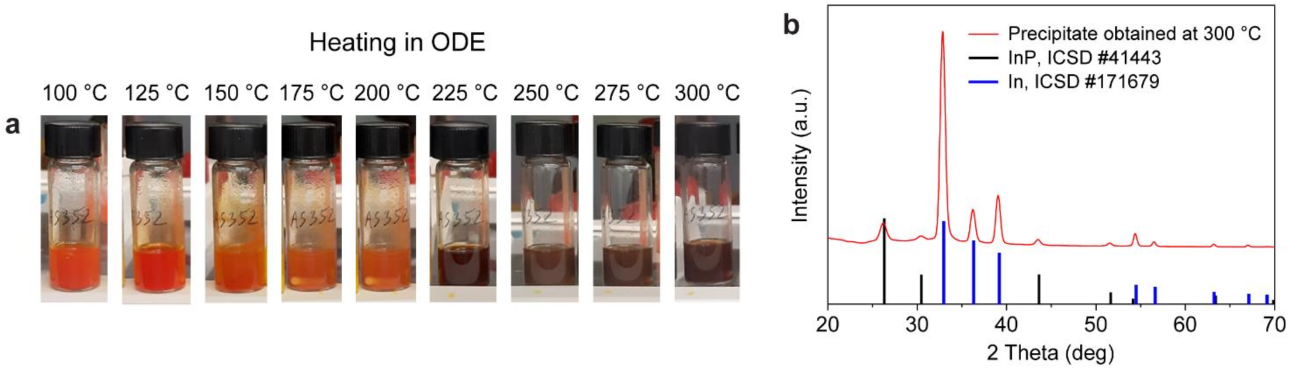

Figure S10. Heating of complex **1** with two equivalents of In[N(TMS)$_2$]$_3$ in ODE (**a**) and powder XRD of the obtained precipitate (**b**).

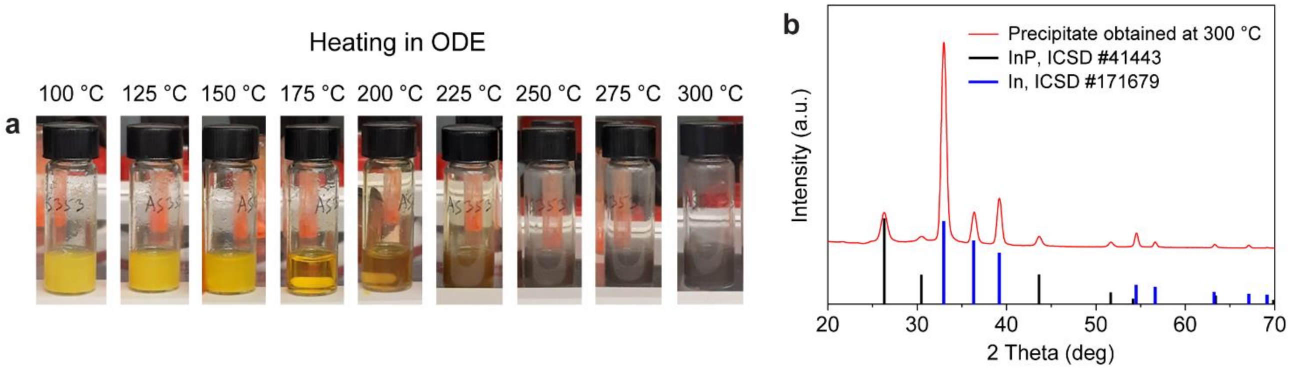

Figure S11. Heating of complex **5** with two equivalents of In[N(TMS)$_2$]$_3$ in ODE (**a**) and powder XRD of the obtained precipitate (**b**).

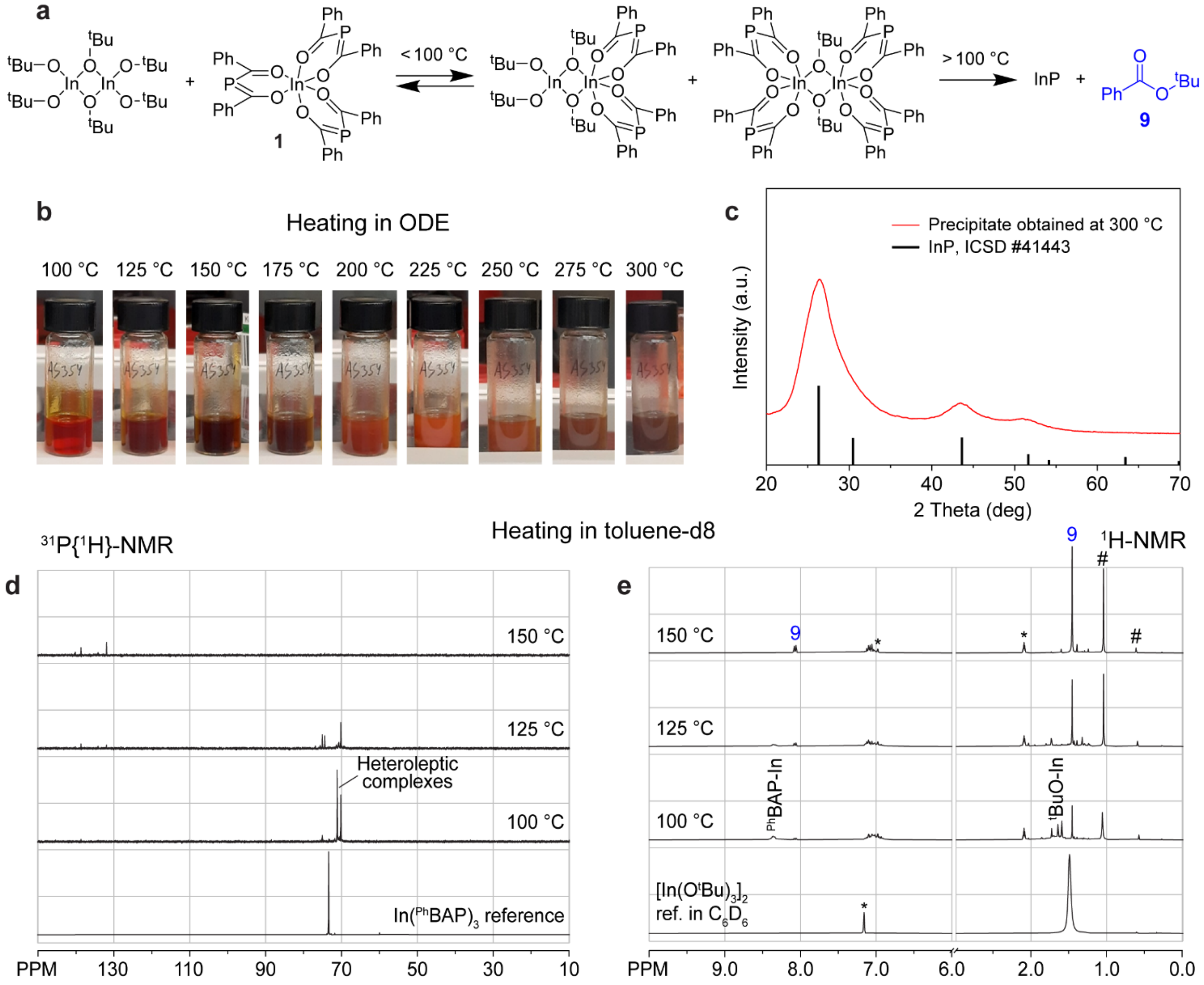

Figure S12. (**a**) Reaction of complex **1** with $[In(O^tBu)_3]_2$ leading to the formation of two different heteroleptic complexes that undergo deacylation above 100 °C with the formation of InP and the organic ester **9**. (**b**,**c**) Visual monitoring of the reaction in ODE (**b**) and powder XRD of the obtained nanocrystalline InP powder (**c**). (**d**,**e**) $^{31}P\{^1H\}$-NMR (**d**) and ^1H-NMR (**e**) of the reaction in toluene-d8. Hash sign (#) denotes ^{t}BuOH impurity that is caused by incomplete dryness of toluene-d8. Asterisk (*) denotes residual solvent peaks.

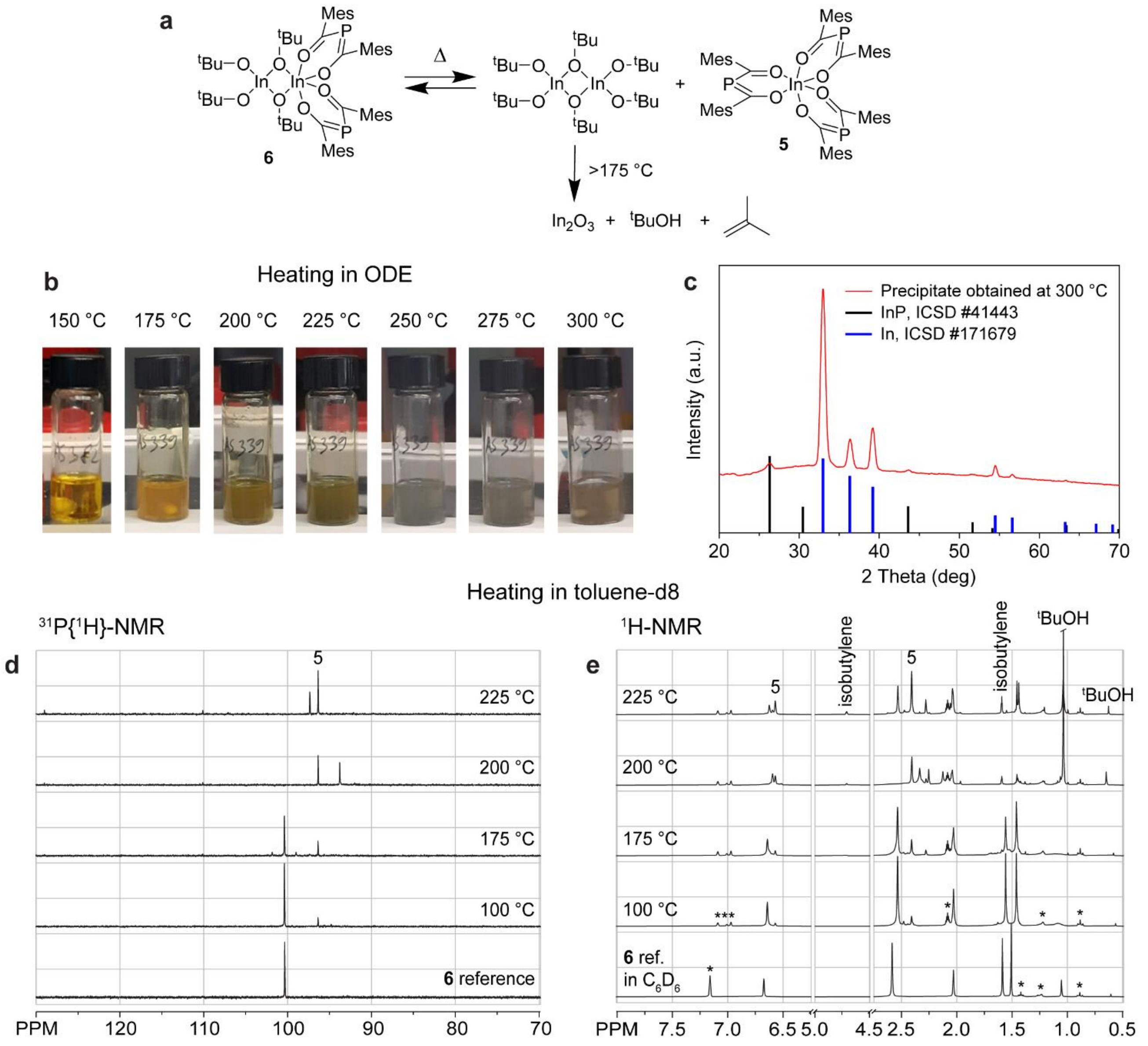

Figure S13. (**a**) Reaction scheme that illustrates the first solvothermal decomposition steps of the heteroleptic complex **6**. Undesired decomposition of *tert*-butoxide is observed above 175 °C. (**b**,**c**) Visual monitoring of the reaction in ODE (**b**) and powder XRD of the precipitate that was obtained at 300 °C (**c**). (**d**,**e**) $^{31}P\{^{1}H\}$-NMR (**d**) and ^{1}H-NMR (**e**) of the reaction in toluene-d8. Asterisk (*) denotes residual solvent peaks.

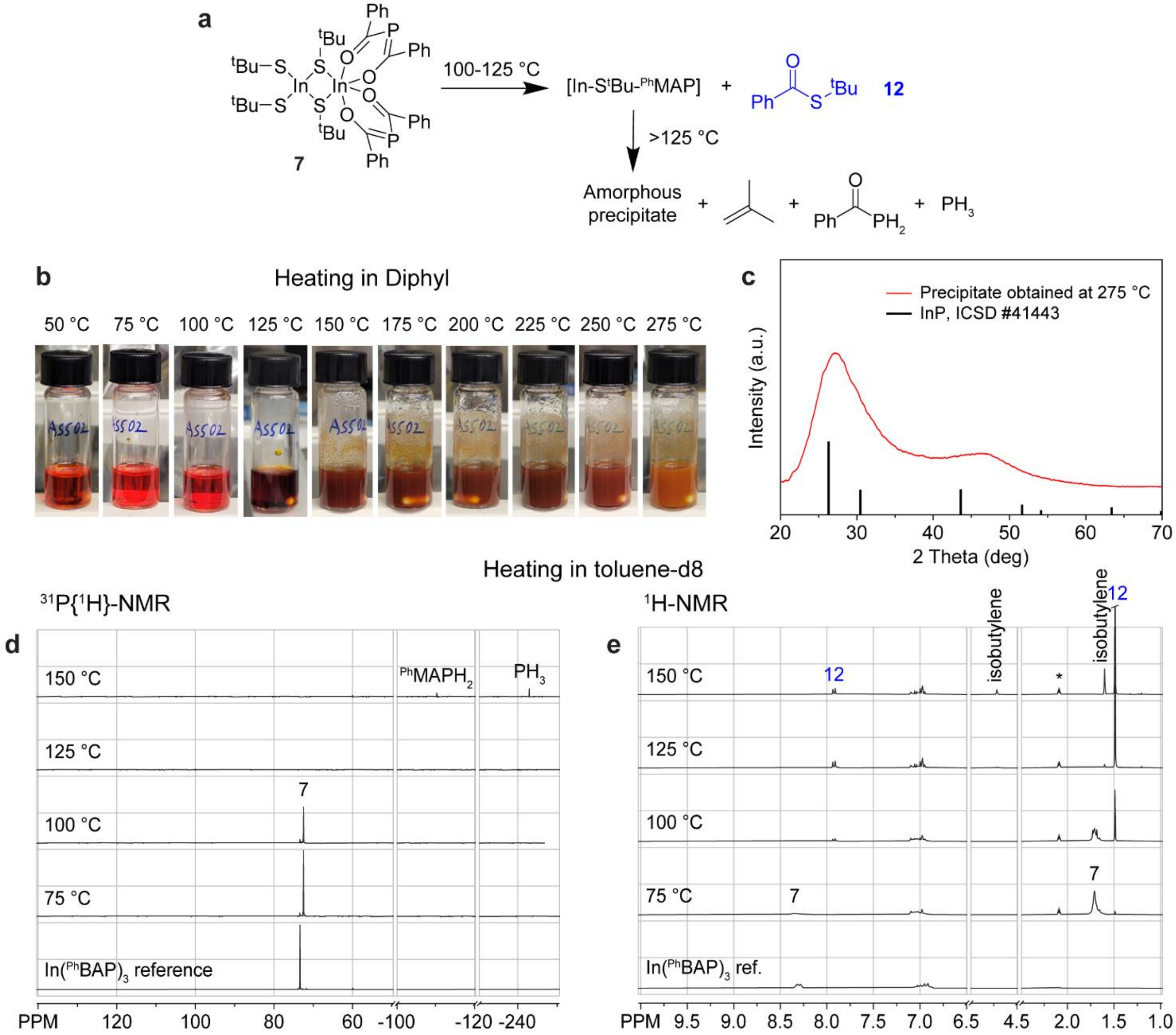

Figure S14. (**a**) Reaction scheme that illustrates solvothermal decomposition of the heteroleptic complex **7**. The first deacylation step is observed between 100 and 125 °C, followed by a competing undesired decomposition of the remaining *tert*-butylthiolate into sulfide, isobutylene and *tert*-butylthiol. *Tert*-butylthiol then reacts with the formed In-^{Ph}MAP intermediate (see **Figure S23**), releasing $^{Ph}MAPH_2$ and phosphine gas. (**b**,**c**) Visual monitoring of the reaction in Diphyl (**b**) and powder XRD of the precipitate that was obtained at 275 °C (**c**). (**d**,**e**) $^{31}P\{^1H\}$-NMR (**d**) and ^1H-NMR (**e**) of the reaction in toluene-d8. Asterisk (*) denotes residual solvent peaks.

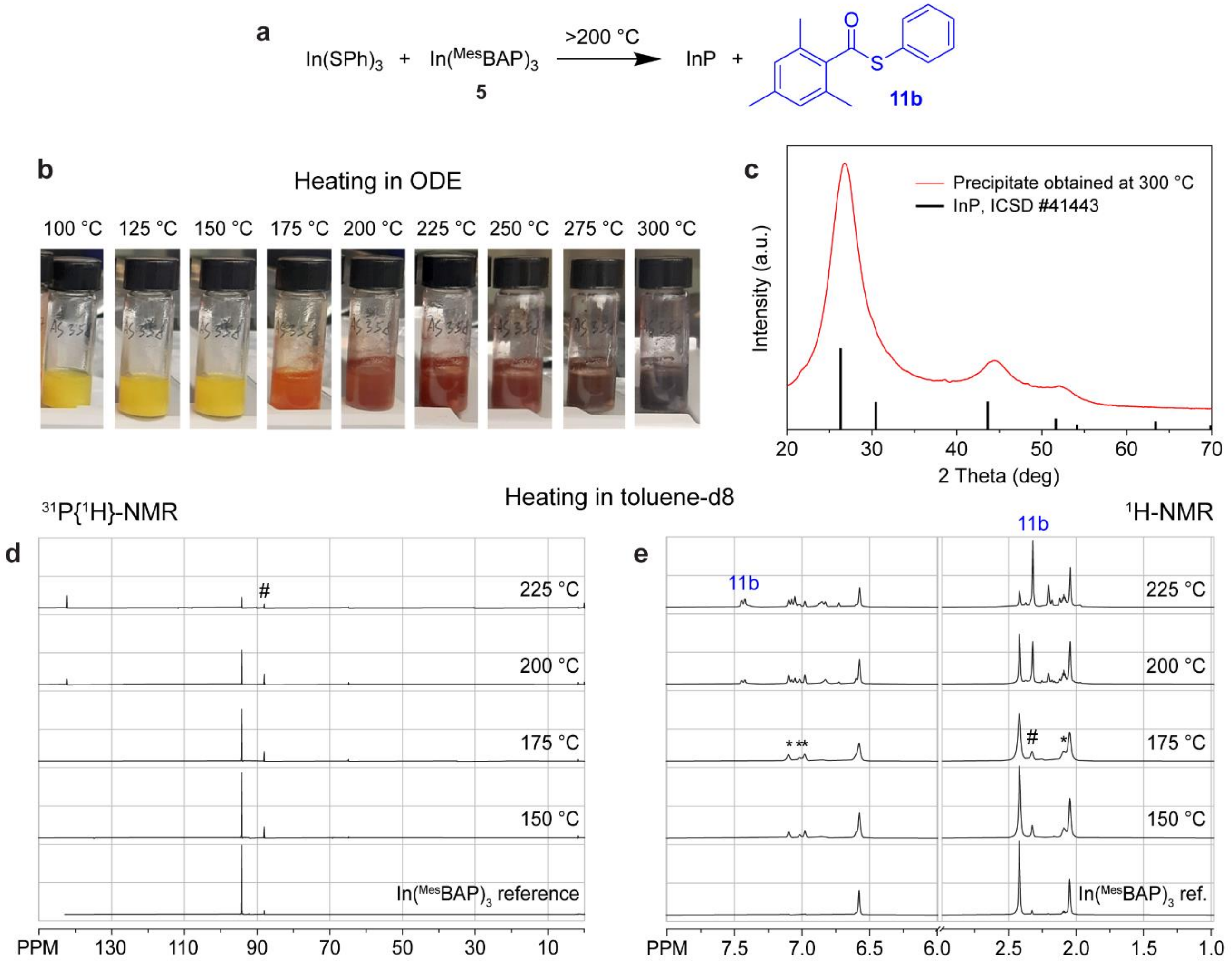

Figure S15. (**a**) Reaction of complex **5** with two equivalents of $In(SPh)_3$ leading to the formation of InP and the organic thioester **11b**. (**b**,**c**) Visual monitoring of the reaction in ODE (**b**) and powder XRD of the obtained nanocrystalline InP powder (**c**). (**d**,**e**) $^{31}P\{^1H\}$-NMR (**d**) and ^1H-NMR (**e**) of the reaction in toluene-d8. Disappearance of the $In(^{Mes}BAP)_3$ signals and appearance of the thioester **11b** signals is observed above 200 °C. Hash sign (#) denotes $^{Mes}BAPH$ impurity that is caused by incomplete dryness of toluene-d8. Asterisk (*) denotes residual solvent peaks. Doublet around 140 ppm in the $^{31}P\{^1H\}$-NMR (**d**) corresponds to a side-product that is formed through a competing $In(^{Mes}BAP)_3$ homolysis.

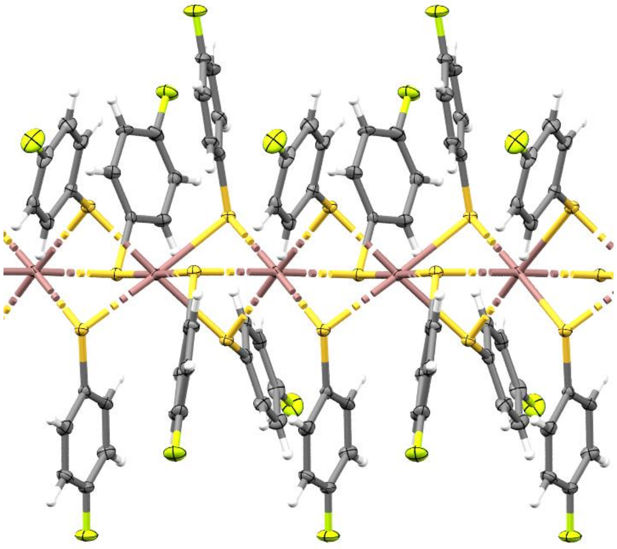

Figure S16. Fragment of the In(SPh-F-*p*)$_3$ crystal structure illustrating a single polymer chain in which indium atoms adopt distorted octahedral geometry.

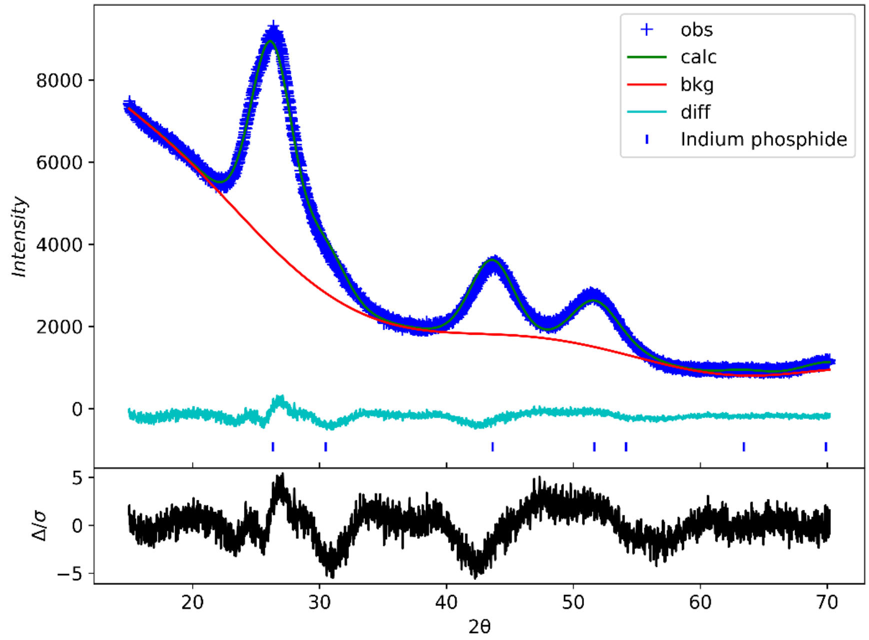

Figure S17. Results of Rietveld refinement for 3.0 nm InP NCs (average size determined from HAADF-STEM). $R_F = 2.43\%$, $R_{F^2} = 3.76\%$, $wR = 3.00\%$. The refined crystallite size is 2.3 ± 0.6 nm (error denotes estimated standard deviation), confirming predominantly single-crystalline nature of the NCs.

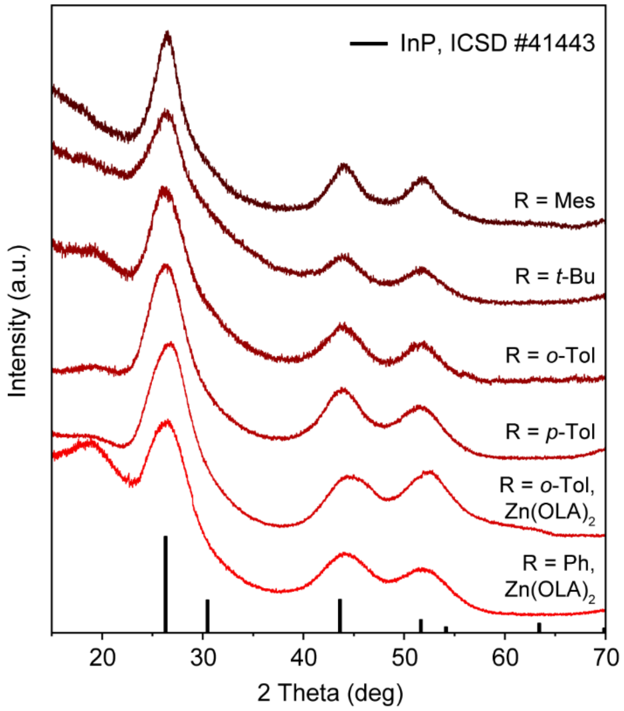

Figure S18. Powder XRD of the isolated InP NCs that are presented in **Figure 4** of the **Main Text**. Note sharpening of InP reflections as the average NCs size increases (from bottom to top). XRD peaks of InP NCs synthesized from complexes **4** (R = *t*-Bu) and **5** (R = Mes) have broad bases, in line with their non-uniform shape/size.

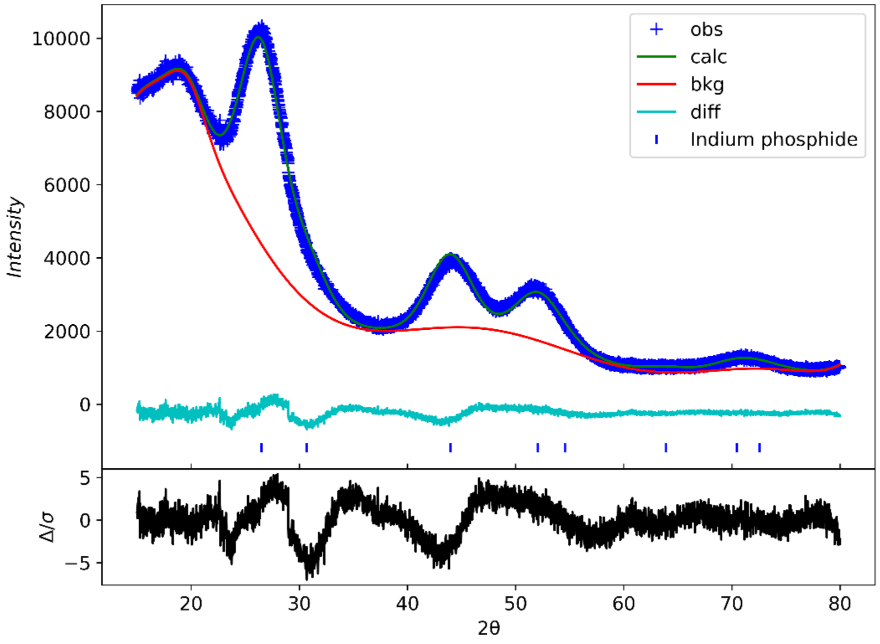

Figure S19. Results of Rietveld refinement for 2.1 nm InP NCs (average size determined from HAADF-STEM). $R_F = 1.80\%$, $R_{F^2} = 3.10\%$, $wR = 3.27\%$. The refined crystallite size is 2.0 ± 0.2 nm (error denotes estimated standard deviation), confirming predominantly single-crystalline nature of the NCs.

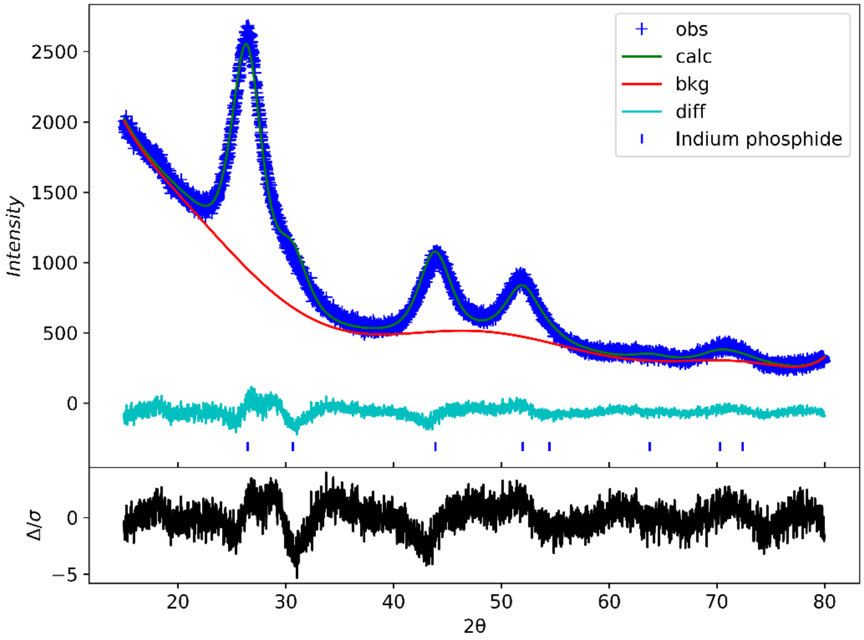

Figure S20. Results of Rietveld refinement for ≈4 nm InP NCs (average size determined from HAADF-STEM). $R_F = 2.30\%$, $R_{F^2} = 3.27\%$, $wR = 4.33\%$. The refined crystallite size is 2.4 ± 0.1 nm (error denotes estimated standard deviation). The discrepancy between the two sizes might be related to the polycrystalline nature of the NCs obtained from complex **5** or to their irregular morphology.

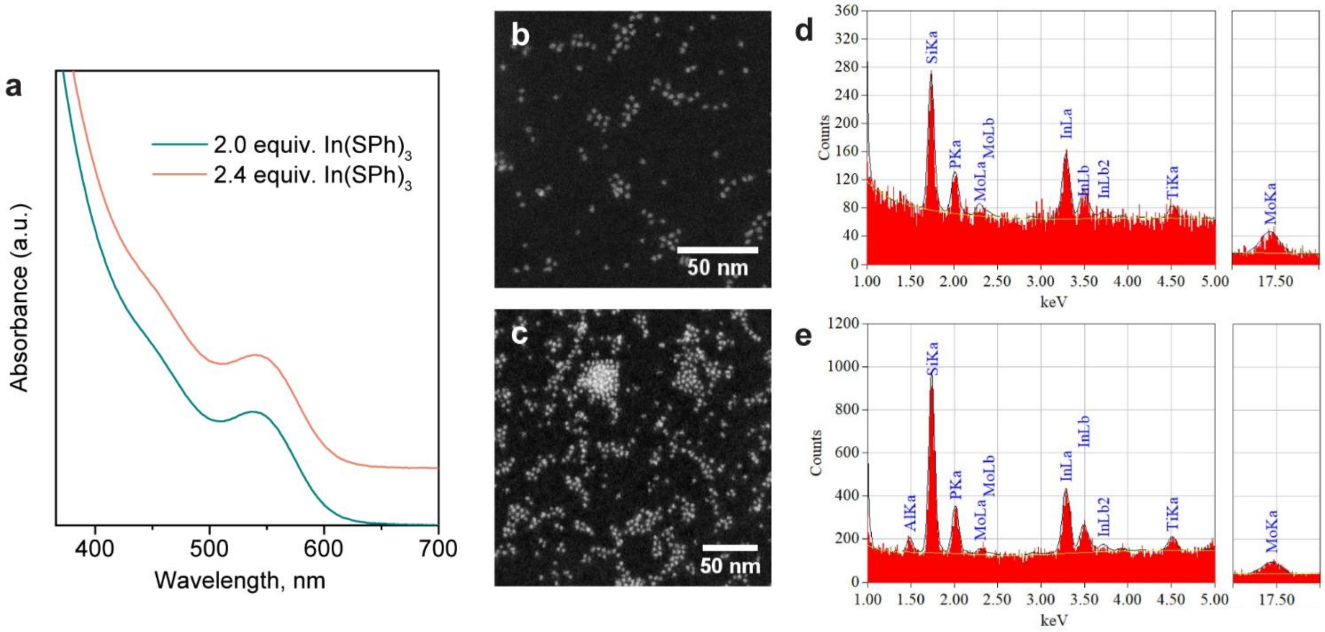

Figure S21. Absorption spectra (**a**), HAADF-STEM images (**b**,**c**) and fitted EDX spectra (**d**,**e**) of InP NCs that were synthesized using a stoichiometric amount of $In(SPh)_3$ (2 equiv., **b**,**d**) and 20% excess of $In(SPh)_3$ (2.4 equiv., **c**,**e**). EDX spectra were acquired from the entire regions that are presented in the panels **b** and **c**. The S Ka line is expected to overlap with the weak Mo La and Mo Lb lines, which originate from the presence of Mo in the instrument parts. The inclusion of S into the fits didn't change their quality, meaning that its content is below our detection limits. Other elements in the EDX spectra (except Si, which can also arise from the silicon grease) originate from the instrument itself and not from the samples of InP NCs.

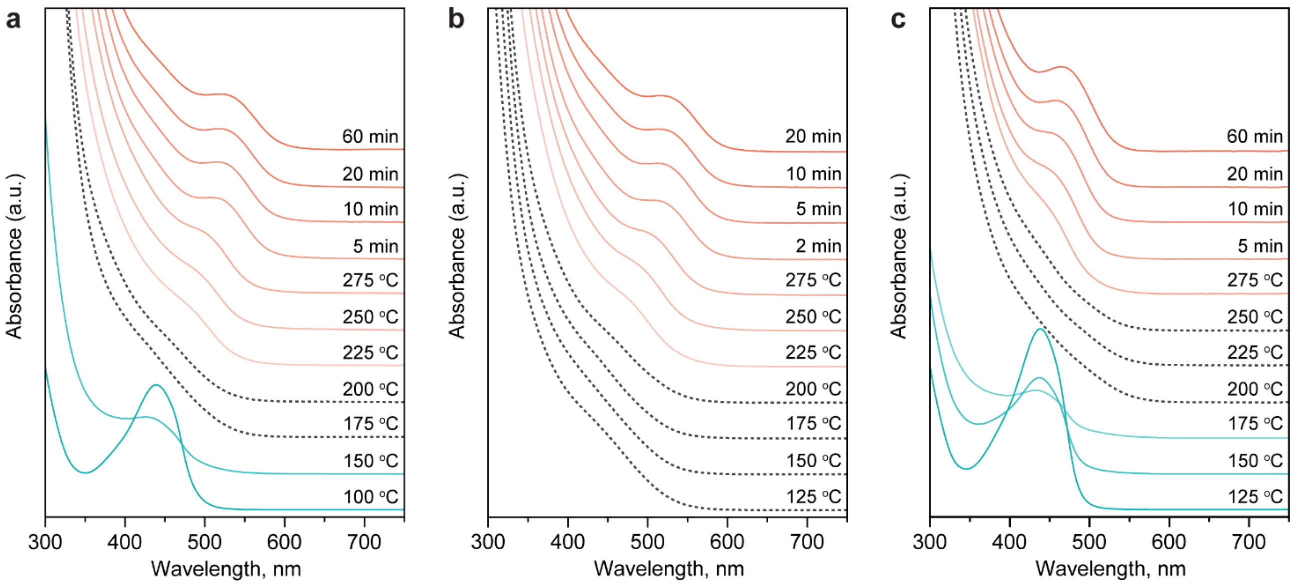

Figure S22. Absorption spectra of reaction aliquots from various versions of the heating-up synthesis. (**a**) Injection of complex **1** into a mixture of $In(SPh)_3$ and $In(OLA)_3$. (**b**) Injection of the In-^{Ph}MAP intermediate into $In(OLA)_3$. In-^{Ph}MAP intermediate was obtained by heating a mixture of complex **1** and $In(SPh)_3$ in Diphyl at 125 °C for 30 min. (**c**) Injection of complex **1** into a mixture of $In(SPh)_3$ and $Zn(OLA)_2$. Absorption spectra of the reaction intermediate (grey dotted lines) appear rather similar, independent of the actual synthesis procedure. Exact quantities of all reagents can be found in the **Experimental Section**.

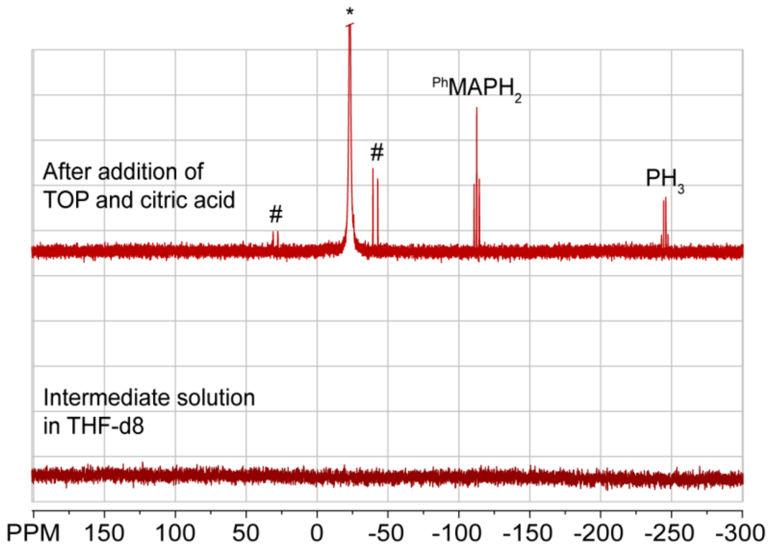

Figure S23. ^{31}P-NMR spectra of the reaction intermediate before and after digestion with citric acid. Asterisk (*) denotes TOP that was added in order to solubilize formed indium citrate. Hash sign (#) denotes unidentified adduct of TOP with a self-decomposition product of In(^{Ph}BAP)$_3$. The preparation of the reaction intermediate (injection solution for hot-injection syntheses) is described in the **Experimental Section**. 0.1 mL aliquot was diluted in 0.4 mL of THF-d8, resulting in a clear wine-red solution with no peaks in ^{31}P-NMR. TOP (50 μL) was added, followed by the addition of excess citric acid, and let to react at room temperature for 12 h, resulting in a clear light-orange solution that contains ^{Ph}MAPH$_2$ and PH_3.

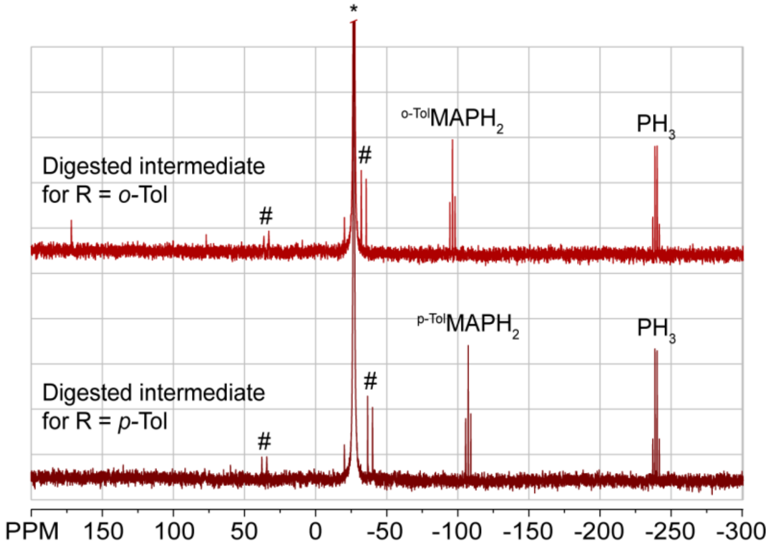

Figure S24. ^{31}P-NMR spectra of the digested intermediates that were prepared from complexes **2** (R = *p*-Tol) and **3** (R = *o*-Tol). Asterisk (*) denotes TOP that was added in order to solubilize formed indium citrate. Hash signs (#) denote unidentified adducts of TOP with the self-decomposition products of In(^{R}BAP)$_3$. Experimental details are analogous to those described in **Figure S23**, except that the digestions lasted for 3 days.

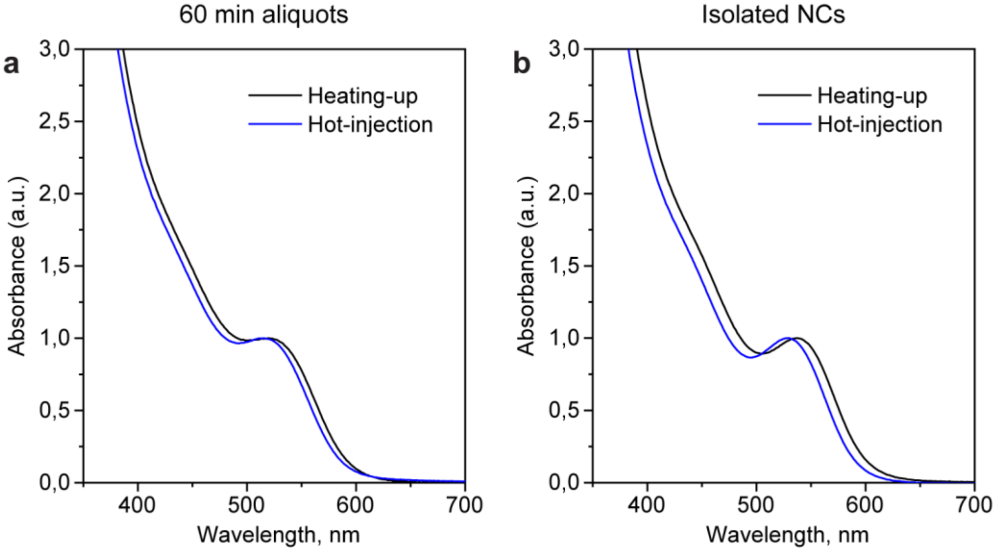

Figure S25. Absorption spectra of the final reaction aliquots (**a**) and the isolated InP NCs (**b**) from the heating-up and the hot-injection syntheses of InP NCs using complex **1**, In(SPh)$_3$ and In(OLA)$_3$ ligand. The hot-injection method produces NCs samples with slightly better-defined excitonic features.

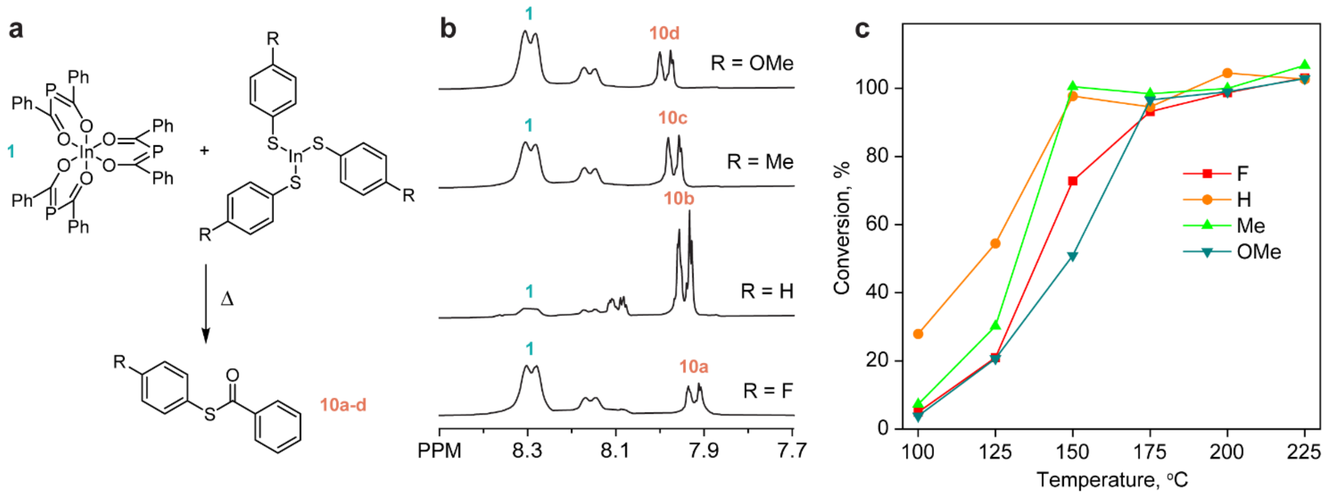

Figure S26. Monitoring of the reaction between complex **1** and various indium arylthiolates in toluene-d8. (**a**) Reaction scheme illustrating the formation of thioesters **10a-d**. (**b**) ^{1}H-NMR spectra (region corresponding to protons in the *ortho*- positions of the benzoyl groups) of the reaction aliquots after 1 hour at 125 °C. (**c**) Conversion as a function of reaction temperature. The conversion was estimated by integrating *ortho*-aryl peaks of the thioesters (labeled in panel **b** as **10a-d**) and normalizing the obtained integrals by the values corresponding to the plateaus in the conversion graphs. Residual $C_6D_5CD_2H$ was used as an internal standard. Reactivity reversal is observed when moving from electron-withdrawing to electron-donating substituents at the aryl thiolate. This is attributed to a combination of two competing effects: an increase in sulfur nucleophilicity that favors the reaction and a strengthening of the In-S bond that needs to be broken in this reaction.

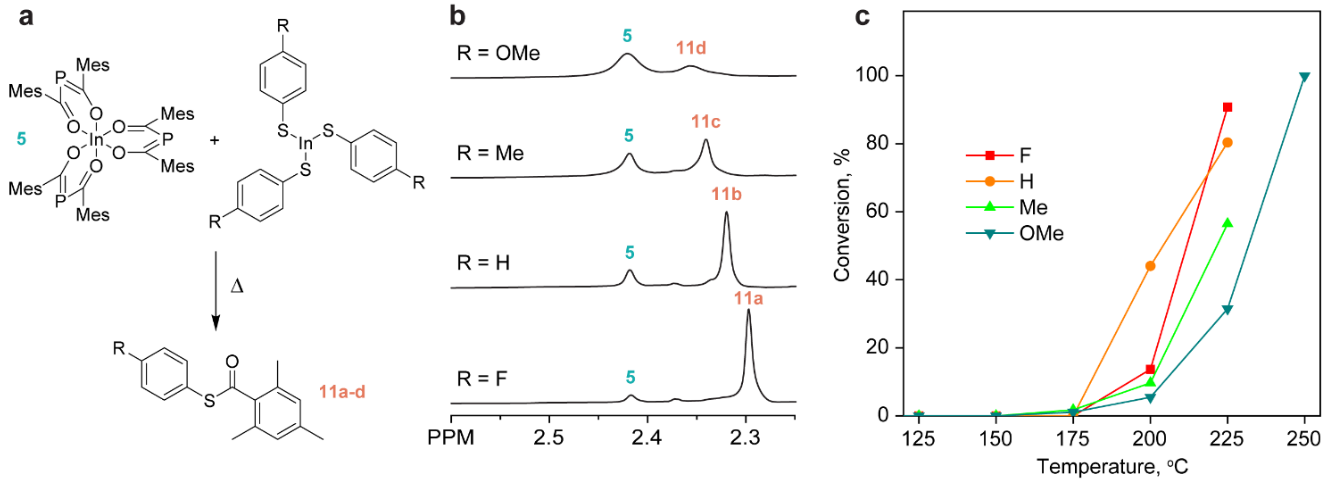

Figure S27. Monitoring of the reaction between complex **5** and various indium arylthiolates in toluene-d8. (**a**) Reaction scheme illustrating the formation of thioesters **11a-d**. (**b**) ^{1}H-NMR spectra (region that corresponds to *ortho*-methyl groups) of the reaction aliquots after 1 hour at 225 °C. (**c**) Conversion as a function of reaction temperature. Conversion was estimated by comparing integrals of the $^{Mes}BAP^{-}$ and the thioester *ortho*-methyl peaks (labeled in panel **b** as **5** and **11a-d**, respectively).

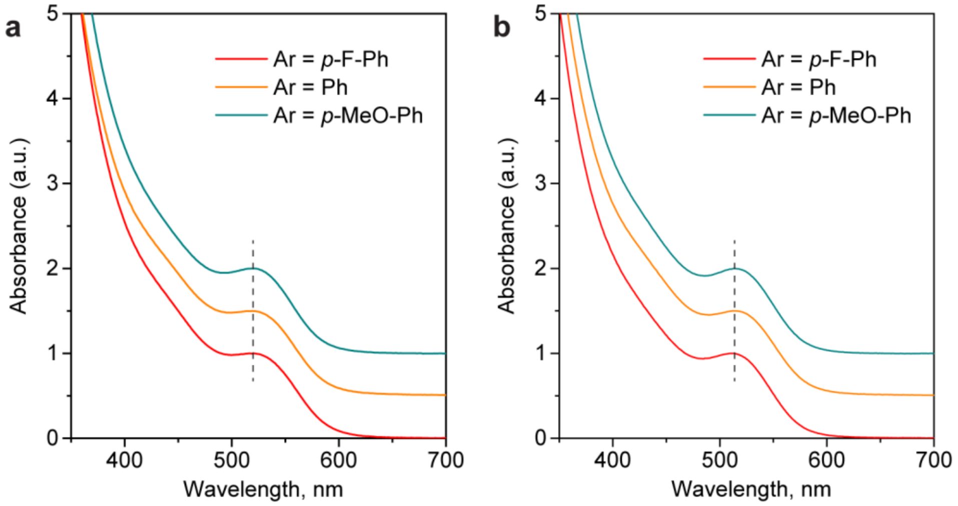

Figure S28. (**a,b**) Absorption spectra of 20 min aliquots from the heating-up (**a**) and the hot-injection (**b**) syntheses of InP NCs by the reaction of complex **1** with different indium arylthiolates, $In(SAr)_3$. In all cases $In(OLA)_3$ was used as a ligand.

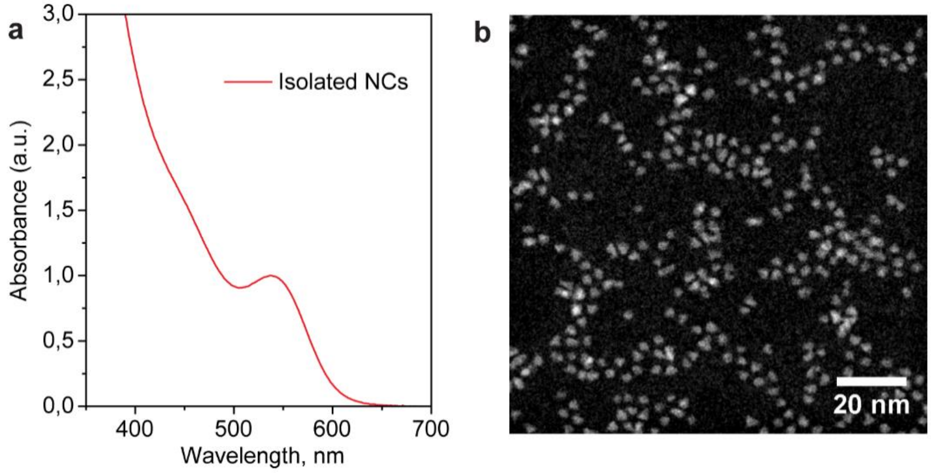

Figure S29. (**a**) Absorption spectrum of InP NCs that were synthesized according to **Route 2** starting from ^{Ph}TAP and $In(SPh)_3$, and using $In(OLA)_3$ as a ligand. (**b**) HAADF-STEM image of the corresponding NCs.

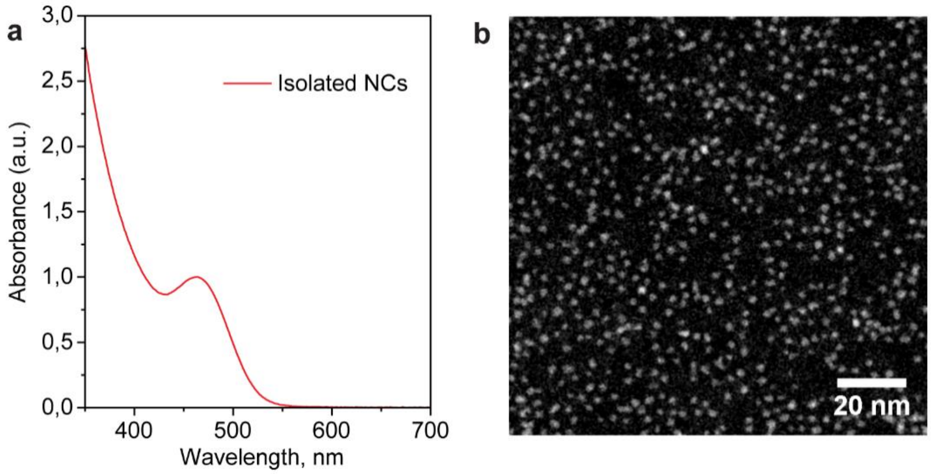

Figure S30. (**a**) Absorption spectrum of InP NCs that were synthesized according to **Route 2** starting from ^{Ph}TAP and $In(SPh)_3$, and using $Zn(OLA)_2$ as a ligand. (**b**) HAADF-STEM image of the corresponding NCs.

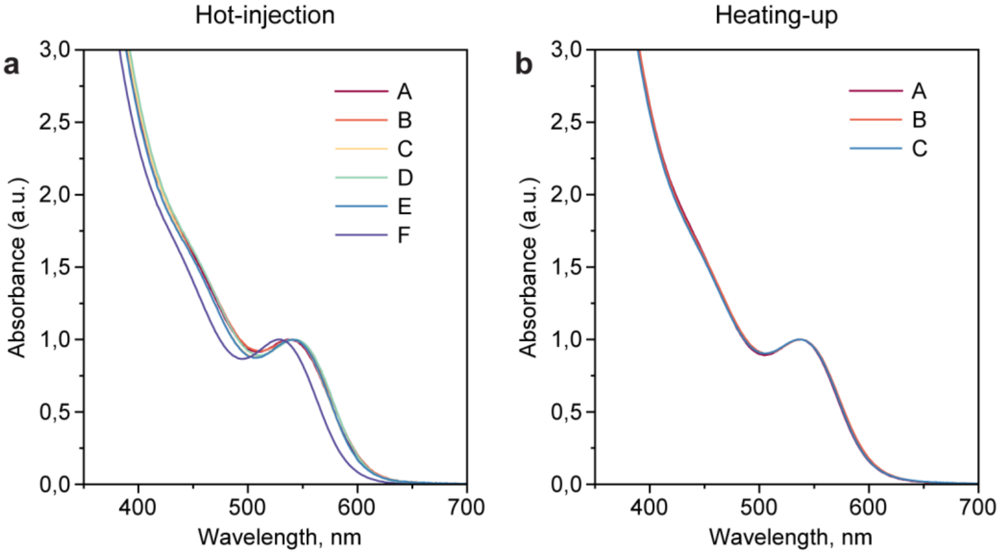

Figure S31. Reproducibility of InP NCs syntheses from complex **1** and $In(SPh)_3$ in the presence of $In(OLA)_3$ ligand. (**a**) Absorption spectra of InP NCs from the hot-injection syntheses with the following variations: A – standard synthesis that is described in the **Experimental Section**; B – 20% excess of $In(SPh)_3$; C – 20% excess of **1**; D – 0.2 mmol (0.83 equiv.) of $In(OLA)_3$; E – 0.16 mmol (0.67 equiv.) of $In(OLA)_3$; F – a different batch of $In(OLA)_3$ (0.1 M). Absorption spectra of InP NCs from the heating-up syntheses with the following variations: A – standard synthesis that is described in the **Experimental Section**; B – reaction at 285 °C; C – $In(My)_3$ instead of $In(OLA)_3$. The syntheses are insensitive to minor variations in the reaction stoichiometry, temperature and the ligand chain length. Instead, specific details of $In(OLA)_3$ preparation seem to have the strongest influence on the average size and uniformity of the resulting InP NCs.

Crystallographic data

Table S2. Crystal data for compounds **1, 2** and **3**.

	**1**	**2**	**3**
Formula	$C_{42}H_{30}InO_6P_3$	$C_{48}H_{42}InO_6P_3$	$C_{48}H_{42}InO_6P_3$
Color	orange-red	orange	orange
Formula weight	838.39	922.54	922.54
Crystal system	Triclinic	Trigonal	Triclinic
Space group / Z	*P-1*, 2	*P-3*, 2	*P-1*, 2
a, Å	10.15090(10)	15.2724(2)	10.63250(10)
b, Å	10.95650(10)	15.2724(2)	10.76770(10)
c, Å	17.7345(2)	10.62550(10)	20.9877(2)
α, °	90.6840(10)	90	94.6760(10)
β, °	95.8710(10)	90	102.5050(10)
γ, °	108.5360(10)	120	110.4050(10)
V, $Å^3$	1858.20(3)	2146.32(6)	2166.02(4)
$\mu(Cu\text{-}K_\alpha)$, mm^{-1}	6.701	5.853	5.799
D_c, g/cm^3	1.498	1.427	1.415
Θ max, °	80.27	80.15	80.13
Meas./Unique refl.	31229 / 7940	10141 / 3085	32597 / 9263
Parameters refined	469	177	556
R1, wR2 [$I > 2\sigma(I)$]	0.0254, 0.0661	0.0270, 0.0698	0.0338, 0.0906
R1, wR2 (all data)	0.0264, 0.0667	0.0287, 0.0707	0.0352, 0.0916
Goof on F^2	1.011	1.050	1.042
Max./min. peak, $e\cdot Å^{-3}$	0.54 / -0.69	0.45 / -0.48	0.98 / -0.78
CCDC Number	2406126	2406125	2406130

Table S3. Crystal data for compounds **4, 5** and **6**.

	**4**	**5**	**6**·0.5Hex·^{t}BuOH
Formula	$C_{30}H_{54}InO_6P_3$	$C_{60}H_{66}InO_6P_3$	$C_{56}H_{80}In_2O_8P_2\cdot 0.5C_6H_{14}\cdot C_4H_{10}O$
Color	yellow	yellow	yellow
Formula weight	718.46	1090.85	1289.98
Crystal system	Monoclinic	Orthorhombic	Triclinic
Space group / Z	$P2_1/c$, 8	$P2_12_12_1$, 12	$P\text{-}1$, 2
a, Å	19.4854(2)	11.82766(8)	13.3062(2)
b, Å	12.50650(10)	20.48574(12)	15.3907(2)
c, Å	31.3185(3)	70.1403(4)	16.8139(2)
α, °	90	90	92.9990(10)
β, °	97.3700(10)	90	101.7300(10)
γ, °	90	90	103.1770(10)
V, $Å^3$	7569.08(12)	16994.88(18)	3265.04(8)
$\mu(Cu\text{-}K_\alpha)$, mm^{-1}	6.468	4.512	6.507
D_c, g/cm^3	1.261	1.279	1.312
Θ max, °	80.30	80.43	75.49
Meas./Unique refl.	24839 / 24839	89953 / 89953	51719 / 13489
Parameters refined	1120	1947	709
R1, wR2 [I > 2σ(I)]	0.0733, 0.2286	0.0351, 0.0937	0.0471, 0.1225
R1, wR2 (all data)	0.0867, 0.2414	0.0360, 0.0942	0.0547, 0.1268
Goof on F^2	1.048	1.068	1.069
Max./min. peak, $e\cdot Å^{-3}$	4.19 / -1.67	0.68 / -0.87	1.16 / -2.00
CCDC Number	2406128	2406133	2406132

Table S4. Crystal data for compounds **7, 8,** and In(SPh-F-*p*)$_3$.

	**7**·0.5Hex·0.5THF	**8**·HNEt$_3$	In(SPh-F-*p*)$_3$
Formula	$C_{44}H_{56}In_2O_4P_2S_4\cdot 0.5C_6H_{14}\cdot 0.5C_4H_8O$	$C_{28}H_{20}Cl_2InO_4P_2\cdot C_6H_{16}N$	$C_{18}H_{12}F_3InS_3$
Color	orange	orange	colorless
Formula weight	1147.84	770.30	496.28
Crystal system	Triclinic	Triclinic	Orthorhombic
Space group / Z	*P-1*, 2	*P-1*, 4	$Pca2_1$, 4
a, Å	11.9390(2)	10.24440(10)	20.5249(4)
b, Å	15.2293(4)	17.69660(10)	12.3792(2)
c, Å	15.5473(2)	19.35870(10)	6.8854(2)
α, °	97.6553(16)	89.0160(10)	90
β, °	97.5744(13)	83.0290(10)	90
γ, °	103.0943(18)	84.1620(10)	90
V, Å^3	2690.11(9)	3465.51(4)	1749.46(7)
μ(Cu-K$_\alpha$), mm^{-1}	9.179	8.046	14.411
D_c, g/cm^3	1.417	1.476	1.884
Θ max, °	74.49	78.00	80.07
Meas./Unique refl.	36890 / 10851	136545 / 14688	7033 / 3133
Parameters refined	616	800	226
R1, wR2 [I > 2σ(I)]	0.0627, 0.1517	0.0295, 0.0726	0.0538, 0.1374
R1, wR2 (all data)	0.0739, 0.1603	0.0305, 0.0734	0.0593, 0.1417
Goof on F^2	1.019	1.082	1.025
Max./min. peak, e·Å^{-3}	1.95 / -0.64	1.42 / -1.07	1.93 / -1.78
CCDC Number	2406129	2406131	2406127

Supporting references

1. Schrader, E. Synthesis and Properties of Acylphosphines. Ph.D. Thesis, ETH Zurich, Switzerland, 2018.
2. Wiesner, T.; Neshchadin, D.; Glotz, G.; Gfader, Z.; Schrader, E.; Christen, S.; Fischer, R. C.; Kelterer, A. M.; Gescheidt, G.; Grutzmacher, H.; Haas, M., Symmetrical and Mixed Tris(acyl)phosphines: Synthesis, Oxidation and Photochemistry. *Chem. - Eur. J.* **2023,** *29* (67), e202302535.
3. Bispinghoff, M. From Elemental Phosphorus to Functionalized Organophosphorus Compounds. Ph.D. Thesis, ETH Zurich, Switzerland, 2017.
4. Becker, G.; Rössler, M.; Uhl, G., Acyl- und Alkylidenphosphane. XX. Bis(2,2-dimethylpropionyl)phosphan und Bis(2,2-dimethylpropionyl)phosphide. *Z. Anorg. Allg. Chem.* **1982,** *495* (1), 73-88.
5. Scott, D. J.; Cammarata, J.; Schimpf, M.; Wolf, R., Synthesis of Monophosphines Directly from White Phosphorus. *Nat. Chem.* **2021,** *13*, 458-464.
6. Bürger, H.; Cichon, J.; Goetze, U.; Wannagat, U.; Wismar, H. J., Beiträgezur Chemie der Silicium-Stickstoff-Verbindungen: CVII. Darstellung, Schwingungsspektren und Normalkoordinatenanalyse von Disilylamiden der 3. Gruppe: $M[N(SiMe_3)_2]_3$ mit M = Al, Ga und In. *J. Organomet. Chem.* **1971,** *33* (1), 1-12.
7. Xu, M.; Jupp, A. R.; Stephan, D. W., Stoichiometric Reactions of CO_2 and Indium-Silylamides and Catalytic Synthesis of Ureas. *Angew. Chem., Int. Ed.* **2017,** *56* (45), 14277-14281.
8. Petrie, M.; Ruhlandt-Senge, K.; Hope, H.; Power, P., Structural Studies of the Monomeric, Low Coordinate, Indium Amides $(t\text{-Bu})_2InN(2,6\text{-}i\text{-}Pr_2C_6H_3)SiPh_3$ and $In\{N(SiMe_3)_2\}_3$. *Bull. Soc. Chim. Fr.* **1993,** *130* (6), 851-855.
9. Andras, M. T.; Hepp, A. F.; Duraj, S. A.; Clark, E. B.; Scheiman, D. A.; Fanwick, P. E.; Hehemann, D. G., Synthesis, Characterization, and Decomposition of the First Mononuclear Eight-Coordinate Indium(III) Benzoate, $In(h^2\text{-}O_2CC_6H_5)_3(4\text{-Mepy})_2$. *Inorg. Chem.* **1993,** *32* (19), 4150-4152.
10. Veith, M.; Hill, S.; Huch, V., Synthese und Charakterisierung von In^{III}–Sn^{II}-Halogenido-Alkoxiden und von Indiumtri-*tert*-butoxid. *Z. Anorg. Allg. Chem.* **2001,** *627* (7), 1495-1504.
11. Suh, S.; Hoffman, D. M., Indium Tris(alkylthiolate) Compounds. *Inorg. Chem.* **1998,** *37* (22), 5823-5826.
12. Briand, G. G.; Davidson, R. J.; Decken, A., Substituent Effects on Indium-Phosphorus Bonding in $(4\text{-}RC_6H_4S)_3In{\cdot}PR'_3$ Adducts (R = H, Me, F; R' = Et, Cy, Ph): A Spectroscopic, Structural, and Thermal Decomposition Study. *Inorg. Chem.* **2005,** *44* (26), 9914-9920.
13. Xie, L.; Shen, Y.; Franke, D.; Sebastian, V.; Bawendi, M. G.; Jensen, K. F., Characterization of Indium Phosphide Quantum Dot Growth Intermediates Using MALDI-TOF Mass Spectrometry. *J. Am. Chem. Soc.* **2016,** *138* (41), 13469-13472.
14. *CrysAlis PRO*, Agilent Technologies Ltd: Yarnton, Oxfordshire, England, 2014.
15. Sheldrick, G. M., Crystal Structure Refinement with SHELXL. *Acta Crystallogr., Sect. C: Struct. Chem.* **2015,** *71* (1), 3-8.
16. Dolomanov, O. V.; Bourhis, L. J.; Gildea, R. J.; Howard, J. A. K.; Puschmann, H., OLEX2: A Complete Structure Solution, Refinement and Analysis Program. *J. Appl. Crystallogr.* **2009,** *42* (2), 339-341.
17. Toby, B. H.; Von Dreele, R. B., GSAS-II: The Genesis of a Modern Open-Source All Purpose Crystallography Software Package. *J. Appl. Crystallogr.* **2013,** *46* (2), 544-549.
18. Calvin, J. J.; Kaufman, T. M.; Sedlak, A. B.; Crook, M. F.; Alivisatos, A. P., Observation of Ordered Organic Capping Ligands on Semiconducting Quantum Dots via Powder X-Ray Diffraction. *Nat. Commun.* **2021,** *12* (1), 2663.
